# Open field trials of food-grade gum in California and Oregon as a behavioral control for *Drosophila suzukii* Matsumura (Diptera: Drosophilidae)

**DOI:** 10.3389/finsc.2023.1141853

**Published:** 2023-05-02

**Authors:** Gabriella Tait, Tingyu Zhu, Jimmy Klick, Fatemeh Ganjisaffar, Claira Castillo, Ryan Kennedy, Hillary Thomas, Christopher Adams, Ferdinand Pfab, Serhan Mermer, Enrico Mirandola, Lan Xue, Frank G. Zalom, Michael Seagraves, Vaughn M. Walton

**Affiliations:** ^1^ Department of Horticulture, Oregon State University, Corvallis, OR, United States; ^2^ Department of Statistics, Oregon State University, Corvallis, OR, United States; ^3^ Driscoll’s Inc., Watsonville, CA, United States; ^4^ Department of Entomology and Nematology, University of California, Davis, Davis, CA, United States; ^5^ Naturipe Berry Growers, Inc., Salinas, CA, United States; ^6^ Department of Horticulture, Mid-Columbia Agricultural Research and Extension Center, Hood River, OR, United States; ^7^ Department of Ecology, Evolution and Marine Biology, University of California, Santa Barbara, Santa Barbara, CA, United States; ^8^ Department of Agronomy, Food, Natural Resources, Animals, and the Environment (DAFNAE), Padova University, Padua, Italy

**Keywords:** spotted-wing drosophila, Integrated Pest Management, behavioral manipulation, pest reduction, field study

## Abstract

The invasion of *Drosophila suzukii*, spotted-wing drosophila, across Europe and the US has led to economic losses for berry and cherry growers, and increased insecticide applications to protect fruit from damage. Commercial production relies heavily on unsustainable use of conventional toxic insecticides. Non-toxic insecticide strategies are necessary to alleviate the disadvantages and non-target impacts of toxic conventional insecticides and improve Integrated Pest Management (IPM). A novel food-grade gum deployed on dispenser pads (GUM dispensers) was evaluated to mitigate *D. suzukii* crop damage in five commercial crops and nine locations. Trials were conducted at a rate of 124 dispensers per hectare in cherry, wine grape, blueberry, raspberry, and blackberry in California and Oregon, USA during 2019 and 2020. The majority of trials with the food-grade gum resulted in a reduction of *D. suzukii* egg laying in susceptible fruit. In some cases, such damage was reduced by up to 78%. Overall, results from our meta-analysis showed highly significant differences between GUM treatments and the untreated control. Modeling simulations suggest a synergistic reduction of *D. suzukii* damage when used in combination with Spinosad (Entrust SC) insecticide. These data illustrate commercial value of this tool as a sustainable alternative to manage *D. suzukii* populations within a systems approach.

## 1 Introduction

Commerce *via* global trade and transport provides a mechanism for introduction of invasive species to new territories, extending pest habitats outside of their native regions (1, 2). Invasive species threaten biodiversity, habitat, nutritious food, clean water, resilient environments, sustainable economies, and human health (3, 4). Agricultural production systems are continuously challenged by invasive species that attack high-value crops, thereby significantly hampering the ability of food industries to maintain profitability (5). The geographic range of agricultural crops provides the potential for invasive species to colonize regions on a global scale (6). Factors that aid expansion include short life cycle, fast growth rate, high plasticity, and resiliency to a wide range of environmental conditions (7). Such factors are drivers of rapid evolutionary change, population increase, and global colonization (2). Practitioners and stakeholders should aim to implement new strategies to manage such new invasive species in agricultural production (8).

*Drosophila suzukii* Matsumura (Diptera: Drosophilidae) is an invasive species native to Southeast Asia. Passive transportation is the main reason of the dispersal of this species (7, 9). It was first detected in North America and Europe in 2008 (10, 11), and later in South America in 2013 (12, 13), and Northern Africa in 2017 (14). The long-serrated ovipositor of *D. suzukii* enables it to oviposit inside fresh fruit, which creates a challenging management problem (15). Emerged larvae burrow within fruit pulp rendering fruit unmarketable (16–19). When *D. suzukii* became established in the U.S. during 2008, the total annual revenue losses for the West Coast berry and cherry industries were estimated at over $500 million (20). Currently the situation is not changed in term of economic impact (17). This particular insect is challenging to manage due to its high dispersal potential, ability to survive and adapt to harsh environmental conditions, and ability to attack a wide host range. For these reasons, *D. suzukii* is a key pest of these fruit industries worldwide.

In the last decade, conventional insecticide uses on affected crops significantly increased to manage *D. suzukii* fruit damage. Typically used insecticides include spinosyns, pyrethroids, and organophosphates (21–23). Intensive use of insecticides poses a tremendous risk to non-target organisms such as pollinators, natural enemies, and humans (24). In addition, frequent insecticide applications likely resulted in resistance development (25, 26). These factors require development of an IPM program that includes alternatives to conventional insecticides for managing *D. suzukii*.

Non-insecticidal control methods including cladding, irrigation, netting, mulching, pruning, monitoring and mass trapping have been implemented against *D. suzukii* (18). While each method provides some relief to *D. suzukii* pressure, they provide limited reductions in crop damage (27). Behavioral control of *D. suzukii* on susceptible fruit (28, 29) indicated promise for industry adoption. The food-grade gum (28, 29) (GUM) possesses tactile and odorant cues resulting in reduced egg infestation. The food grade gum makes use of physical properties to mimic fruit, resulting in *D. suzukii* laying their eggs in a soft gel-like substrate, instead of the fruit itself. The food grade gum is a mixture of food-grade ingredients which is highly attractive to *D. suzukii* and competes with the ripening fruit throughout the season (28, 29). To the best of our knowledge, the food-grade gum modifies various *D. suzukii* behaviors, ultimately resulting in a significant decrease in fruit damage. The product diverts *D. suzukii* away from ripening fruit, which results in significant retention of the pest, keeping it away from fruit. Third, the food-grade gum acts as an egg sink. Since the *D. suzukii* eggs laid in this medium cannot develop, this translates in a substantial reduction of the pest population growth (28, 29).

The aim of this work was to determine the potential of the food-grade gum to reduce *D. suzukii* damage in large-scale commercial open-field and screenhouse fruit production units on blueberry, cherry, raspberry, blackberry, and wine grape. The hypothesis was that food-grade gum would reduce *D. suzukii* damage in small fruit, tree fruit and grapes under semi-field and small-scale field conditions.

These studies were conducted during 2019 and 2020 in California (Salinas, Santa Maria, Oxnard, and Watsonville) and Oregon (Corvallis, Hood River, Independence, Yamill, and Riverbend) in the western United States.

## 2 Materials and methods

In all field trials, GUM dispensers were placed at least 27 meters away from untreated control (UTC) plots to minimize volatile plume interaction between treatments. In the current study, cotton pads (2.5x1.5 cm Cotton Ovals, Swisspers, Gastonia, NC, USA) were used to apply ~1.8 g of GUM on each dispenser at the rate of 124 dispensers per hectare under commercial production conditions (28). Cotton pads were placed directly on the ground close to irrigation drippers to provide adequate daily moisture. Earlier work illustrated that dispensers have a field longevity of 21 days and for this reason, dispensers were therefore deployed 1 to 4 times depending on the duration of crop ripening and susceptibility.

In three trials (Wine grape 2019, Cherry 2019, and Blueberry 2020 Trial 2), egg laying data were collected in buffer plots that were located between UTC and GUM plots to determine the active range of released volatiles beyond treated areas. This design was implemented based on the assumption that volatiles from treatment plots may be blown or diffuse beyond treatment plots. Berries were brought to the laboratory to determine number of eggs in fruit for each of the plots using a dissecting microscope. All soft or damaged fruits were excluded when assessing presence of eggs.

In some cases, at first fruit color, laboratory-reared *D. suzukii* flies were released in each plot with the intent to create a relatively even pest pressure in all plots. Colonies of *D. suzukii* used in field studies consisted of seasonally collected wild adults from multiple field sites in the Willamette Valley, Oregon, and Oxnard, California. Collected adults were released into plastic cages and reared at 24°C and 70% relative humidity, with a 16:8 (L:D) h photoperiod before being released in the respective field trials. Flies were constantly provided with water and artificial diet (30) that served as both a food source and an oviposition medium. Before their use in experiments, all flies were allowed to mate for 8 d in mixed-sex cages. Some small fruit varieties were numbered since this information is proprietary.

### 2.1 Oregon

#### 2.1.1 Wine grape (2019)

A replicated field trial on drip-irrigated Pinot noir winegrape (*Vitis vinifera* L.) was conducted in Yamhill County, Oregon, USA (45°6′59″N 123°12′20″W) from 10 to 18 October 2019 on ~2.6 hectares. Vines were spaced at 1.5 by 5 m, and trellised on a standard four wire trellis system, supporting a ~2 m canopy. Rows were oriented along a north-south direction on an east facing slope. Three treatments (i.e., UTC, buffer, and GUM), were included with ~0.056 ha plots. No pesticides were applied during the experimental period. Here, there were 28 GUM and buffer plots each and 18 UTC plots. GUM dispensers were applied on 10 October and ten berries were collected from each plot on this date. Sampling dates were 11, 14, and 18 October 2019.

#### 2.1.2 Cherry (2019)

Trials were conducted in a commercial sweet cherry (*Prunus avium* L.) orchard located at the Mid-Columbia Agricultural Research and Extension Center (45°68’515’’N, 121°51’67’’W), Hood River, Oregon, USA. A 1.12-hectare orchard was divided into twelve plots (~0.07 hectare, ~41 trees per plot, cultivar Regina). UTC, buffer, and GUM plots were replicated four times. The GUM dispensers were deployed on day 0 (16 June) (8 dispensers per plot). No insecticides were applied to the orchard for the duration of the experiment. Here, an additional 200 mated 8- to 12-day-old *D. suzukii* were released in the center of each plot on a weekly basis on 23 June, and 1, 8, and 15 July 2020 (800 total). Data were collected for 35 days from 16 June through 22 July 2019. Because of relatively large canopy size of cherry trees compared to the other crops, ten cherries were collected from the lower (0.9 m), middle (1.5 m), and upper (2.1 m) portions of the central two trees in each plot (30 per tree, 60 total per plot) weekly.

### 2.2 Blueberry (2020)

#### 2.2.1 Trial 1

The trial was conducted in an organic highbush blueberry (*Vaccinium corymbosum* L.) (cultivar 1) planting on 23.76 hectares in Independence, Oregon, USA (44°51′11″N 123°11′29″W). The experiment began on 9 July and continued through 11 September 2020. There were three treatments: UTC (no insecticide or GUM applications), grower standard (GS, insecticide application), and GUM. Grower standard applications targeting *D. suzukii* at the registered field rate included spinosad (Dow AgroSciences LLC, Indianapolis, IN) (Entrust, 454 L water and 70 g Spinosad; 13 July, and 7 September), peroxyacetic acid (Jet-Ag 5%, 454 L water and 253 ml; 17, 20, 23, 28 July, 3, 10, 17, 24 August and 11 September) and *Chromobacterium subtsugae* (Marrone^®^ Bio Innovations, Davis, CA) (Grandevo, 454 L water and 21.5 kg; 22 July, 7, 13, 21 August and 3 September). Each treatment had 12 plots, and each plot was ~0.66 hectare. GUM dispensers were deployed on 9 and 13 July (81 dispensers per plot), and on 9 and 22 August 2019. Blueberries were collected every two days for the duration of the experiment. One fruit sample (each sample consisted of 10 berries) was collected in each of the respective 36 plots. Collected samples were at least 20 m from the edge of the crop and each sample contained 10 blueberries collected from the interior of the bush at ~0.75 m from the ground.

#### 2.2.2 Trial 2

Trials were conducted in 1.8 hectare of highbush blueberry plants (Independence, Oregon, USA, 44°51′11″N 123°11′29″W) (cultivar 2). The experiment ran from 6 October to 15 October 2020. There were three treatment levels i.e., UTC (pesticide application), buffer (area with no treatments between the UTC and the GUM to determine active distance of volatile impacts on *D. suzukii*), and GUM. The GUM plots were located directly next to the buffer, followed by UTC plots of equal size. Plots were each ~0.05 hectares (12 plots per treatment). Spinosad (see rates above)was applied on 6 October on the UTC and buffer areas. Insecticide application and GUM deployment (6 dispensers per plot) occurred only on 6 October. On 8, 10, 13 and 15 October, one fruit sample consisting of 10 berries was collected from each of the 36 plots. Samples were collected at least 20 m from the edge of the crop and at ~0.75 m above the ground.

### 2.3 California

#### 2.3.1 Blueberry screenhouse (2020)

This trial was conducted in Oxnard, California, USA (34.1975° N, 119.1771° W) on highbush blueberry (cultivar 3) plots during 2020. Plants were irrigated with three drip stakes per plot ten times a day for ten-minute intervals delivering 1.1 liters of water per hour. Screenhouses were fully enclosed with screen material to prevent insects from entering. There were three 70 m x 5 m screenhouses with GUM or UTC treatment randomly assigned to the north or south end of each screenhouse for a total of 6 plots. Within each screenhouse, treatment plots contained twelve plants in two rows, and plots were separated by 45 m. one-hundred flies (8-10 day-old) were released in each plot four times, once per week. Three GUM deployment plots were compared with three UTC plots. GUM dispensers were installed in every other plant with irrigation stakes placed directly through the pads. The GUM application was completed on 14 April. Plots were sampled every seven days from 14 April to 12 May. One sample consisted of 50 berries.

#### 2.3.2 Raspberry, blackberry, and strawberry (2020)

Ten field trials were conducted from September to November 2020 across multiple coastal production regions in California, USA (Watsonville, Salinas, Santa Maria, Oxnard), at different ranches and on multiple varieties (i.e., cultivar 4, cultivar 5, cultivar 6 for raspberry; cultivar 7 for blackberry; cultivar 8 for strawberry) being grown under high tunnels. Each location was a replicate consisting of two plots (0.4 to 2 ha) and were randomly assigned at each ranch to GUM or to UTC. Plots within a ranch received similar irrigation, fertilizer, and insecticides. Each plot received a minimum of four spinosad (see rates above) sprays timed 7-10 days apart during the cropping period and based on monitoring trends from fruit collections. Additional peroxyacetic acid applications (see rates above) were applied at 2-3 day intervals after each spinosad application, followed by a *C. subtsugae* application 1-2 d after each peroxyacetic acid application. Throughout the experimental periods, GUM dispensers were distributed evenly throughout each plot and replaced every 21 days. GUM dispensers were staked directly under the drip line in soil plots, and irrigation stakes were placed directly through the dispenser in substrate plantings. Six fruit samples were collected from each treatment plot every week for 4 to 12 weeks. Samples were collected at least 2 m from each edge of the tunnel as well as from the center of the tunnel approximately 20-30 m from the edge of the tunnel and at ~0.75 m from the ground. Each sample consisted of 50 berries. Sample berries were incubated at room temperature for 2-4 days to allow for larval growth and facilitate detection. Samples were evaluated by crushing fruit and submerging them in a saltwater solution (30). The crushed fruit solution was then poured into a tray where *D. suzukii* larvae subsequently floated to the top of the solution and were counted.

#### 2.3.3 Blackberry (2020)

A trial was conducted in a 4.85 ha blackberry field with cultivar Prime-Ark^®^ 45, in Salinas, California, USA in 2020 (36.6777° N, 121.6555° W). Each treatment consisted of 7 plots: UTC, (GS, standard insecticide application), GUM, or GS + GUM. Each plot was approximately 0.23 hectares. Two insecticide treatments (zeta-cypermethrin, Mustang Maxx^®^, FMC Corporation, Philadelphia, PA, USA) were applied on 19 and 25 September in the GS and GS + GUM plots. The GUM dispensers were deployed on 21 September 2020, three days before the first sampling, and reapplied on 8 October 2020 in the GUM and GS + GUM treatments. The gum was placed under drip emitters. Blackberry samples were collected twice per week with one pre-treatment collection (11 September 2020) occurring before the insecticide treatments and gum deployment. Each sample consisted of 300 blackberries for each plot. Biweekly collections continued for 5 weeks after the pre-treatment count. Samples were evaluated 3 to 4 days after collection to allow larvae to develop and the number of *D. suzukii* eggs and larvae were counted under a stereo-microscope (31).

## 3 Data analyses

For the Oregon winegrape where the number of eggs was collected and the California raspberry, blackberry, strawberry fields, and blackberry open-field where the number of larvae was collected, the mean number of eggs or larvae per berry (denoted as $m=\frac{\#eggs(larvae)}{\#berries}$ ) on each sampling date at each plot was considered as the variable of interest. The log transformation *log (m + 0.05)* was applied where 0.05 was added to accommodate the zero counts of eggs.

To account for the differences in initial conditions of each plot and assess the relative effectiveness of the treatment effects based on how they performed in reducing the number of eggs or larvae over time, the analyses focused on the change in the log transformed mean number of eggs or larvae per berry between sampling date *j* and the start date *1* with Δ*m_j_ = log(m _j_ + 0.05) – log (m_1_ + 0.05)* for *j=*2,3,… at each plot. A negative Δ*m_j_* value indicated that the mean number of eggs or larvae per berry at sampling date *j* is lower than that at the start date 1, and vice versa. A linear mixed model (PROC MIXED in SAS 9.4) was used to analyze Δ*m_j_*, where treatment, sampling date, the interaction between treatment and sampling date and plot were fixed effects. The REPEATED statement in PROC MIXED was used to account for possible correlation among repeated measurements on the same plot at different sampling dates. When the interaction between treatment and sampling date was not significant, Tukey-Kramer’s Honest Significant Differences test was used to compare pairwise differences among the treatment means. However, when the interaction was significant (P < 0.05), interpreting the main effect of treatment can be misleading. Thus, the SLICE statement in PROC MIXED was used to generate the simple effects analysis, which compared differences among treatments within each sampling date.

For Oregon blueberry cultivar 1, egg counts were mostly zero. Among 864 total records of three treatment levels, no eggs were found in 825 records, while eggs were reported only in 39 records. To address the excessive zeros in the data, a dummy variable was created to indicate the existence of eggs, and a logistic regression (PROC LOGISTIC in SAS 9.4) was used to model the infestation rate for each plot, namely the probability of each plot having eggs. In the logistic regression model, treatment, sampling date, treatment and sampling date interaction, and quadratic term of sampling date were included as explanatory variables. The quadratic date effect was included since it provided a better model fit with smaller AIC/BIC value. The sampling date and its quadratic term were treated as continuous variables instead of fixed effects. This was because there were 24 sampling dates in the experiment and treating it as a fixed effect would increase model complexity and result in a slightly overfitted model according to Akaike Information Criterion (AIC). A random effect was not included since the mixed logistic model failed to provide reliable inference due to the excess zeros in the egg counts. Furthermore, a firth correction (32) was specified using FIRTH option in the MODEL statement to account for the imbalance of zero and nonzero counts observed in the data.

The infestation rate was considered for trials that contain number of infested berries, namely Oregon winegrape, cherry, blueberry cultivar 2, and California screenhouse (blueberry cultivar 3). The egg infestation rate (r) was defined as the number of berries containing eggs divided by the total number of berries collected on each sampling date at each plot ( $r=\frac{\#infestedberries}{\#berriescollected}$ ). Resulting infestation rates were then subjected to arcsine-squared root transformation defined as $arcsin\;(\sqrt{r})$ . Same as the analysis of the number of eggs, the change in infestation rate over time was considered, defined as $\Delta r_{j}=arcin(\sqrt{r_{j}})-arcsin(\sqrt{r})$ for *j* = 2, 3,… at each plot. A negative Δ^*^_j_ indicates that the infestation rate at sampling date *j* is lower than that at the start date 1, and vice versa. A linear mixed model (PROC MIXED in SAS 9.4) was used to model *Δr_j_*, where treatment, sampling date, the interaction of treatment and sampling date and plot were fixed effects. The REPEATED statement in PROC MIXED was used to account for possible correlation among repeated measurements on the same plot at different sampling dates.

Multiple independent trials were conducted to test the treatment effect. To effectively combine results from multiple analysis and increase the generalizability of the analysis results, we employed Stouffer’s method (33) to combine a total of 10 p-values from trials testing the difference in mean number of larvae in terms of GUM vs UTC, specifically those from California tails, including raspberry, blackberry, and strawberry (2020). The null hypothesis for the meta-analysis is that all of the individual null hypotheses are true, indicating no significant difference between GUM and UTC, while the alternative hypothesis is that at least one of the individual alternative hypotheses is true. The test was implemented using the “stouffer” function in the R package “poolr.

## 4 Population modeling

The buildup of *D. suzukii* populations was modeled under four scenarios i.e.; no intervention (UTC), GUM only, insecticide (GS), and GUM and insecticide (GS + GUM). The model parameters were obtained from experimental work (34–37) and iterations of the model have been used in previous studies (36). Recorded *D. suzukii* population levels and weather data were used as model inputs. Outputs from the model were directly compared with *D. suzukii* infestation data of the blueberry field trial 1 (2020, in Oregon). This trial was selected because of its relatively long duration and is most suitable for describing population build-up.

Ambient temperature influences the fecundity rates, mortality rates, and maturation delays of the four principal life stages (eggs, larvae, pupae, and adults). The simulations were based on daily mean temperature data recorded at Aurora, Oregon, USA, between June and September 2020. We assumed that the flies had access to unlimited fruit (blueberry) and that no other factors affected population dynamics (e.g., no immigration or emigration, no predators or parasitoids, and no effect of humidity). Parameter values, including for fecundity rates, mortality rates, and maturation delays, were obtained from laboratory experiments on blueberry (34–36). The simulations were initialized on 30 June with a population composed equally of adult males and females. The model simulations track relative population densities, and the initial adult density was chosen so that the simulated egg density matched the final eggs/berry in the UTC treatment. The GUM dispensers were assumed to reduce *D. suzukii* fecundity by 49%, according to data from Tait et al. (29). Insecticide induced mortality rates caused by GS (spinosad) were calculated from laboratory data (21). Spinosad was used as insecticide model. The effects of GUM and GS were assumed to start on 9 July in accordance with the model design. Details on the model and on how the GS and GUM treatments were implemented can be found in the **Supplementary Material**. The simulations were implemented using Wolfram Mathematica 13.0 (38). The code for the simulations is available online^1^.

## 5 Results

### 5.1 Oregon

#### 5.1.1 Wine grape (2019)

**Figure 1:**
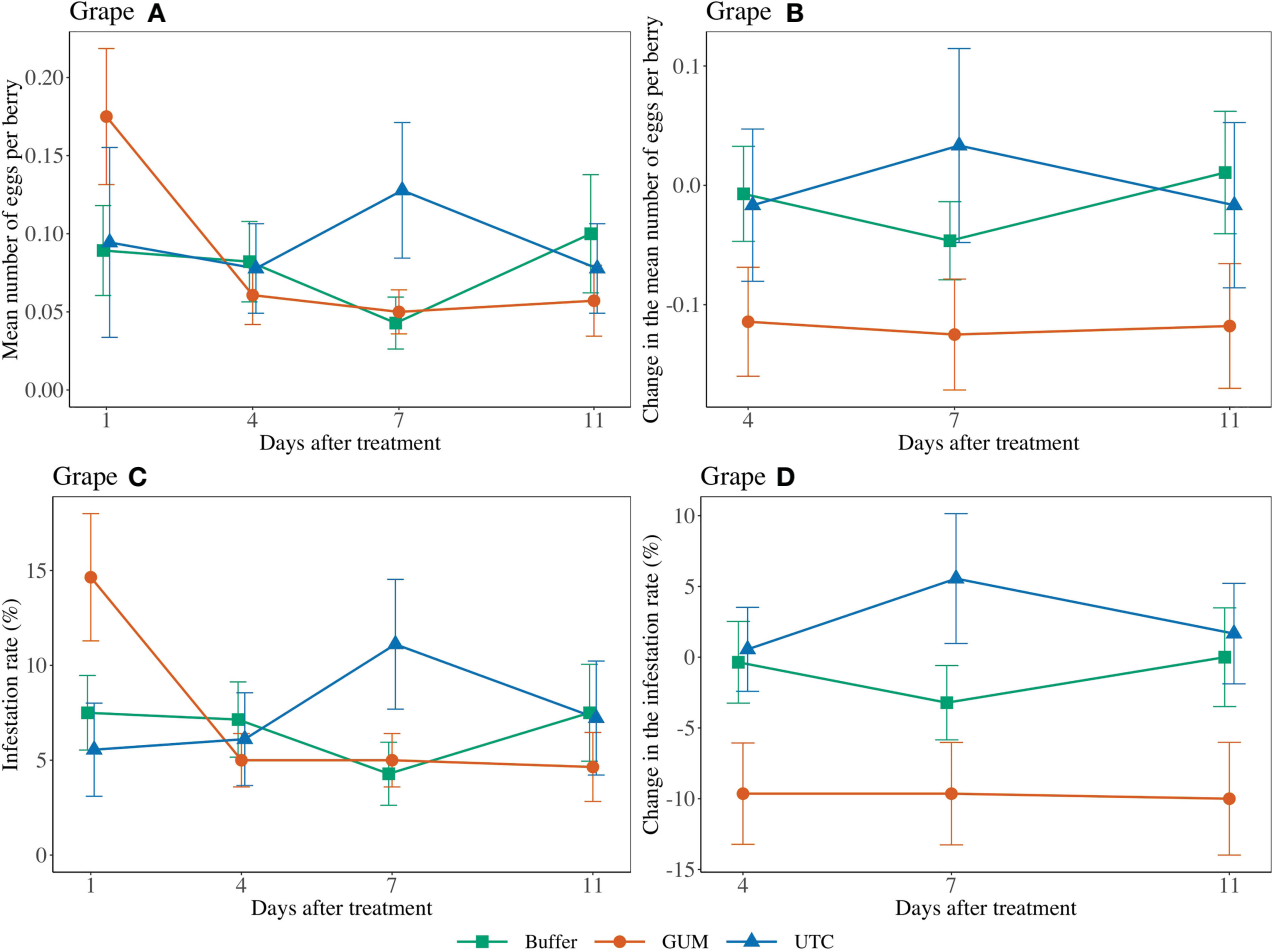
Effect of food-grade gum treatments on the mean number of *D. suzukii* egg per berry and egg infestation rate (± SEM) on Pinot noir in the Willamette Valley in Oregon during 2019. The sample mean and SE of egg numbers per berry for GUM, UTC, and buffer were 0.086 ± 0.014, 0.094 ± 0.021, and 0.079 ± 0.014, respectively **(A)**. The sample mean and SE of changes in the number of eggs per berry for GUM, UTC, and Buffer were -0.119 ± 0.027, 0.000 ± 0.041, and -0.014 ± 0.024, respectively **(B)**. The sample mean and SE of the infestation rate for GUM, UTC, and Buffer were 0.073 ± 0.011, 0.075 ± 0.014, and 0.066 ± 0.010 **(C)**. The sample mean and SE of changes in infestation rate for GUM, UTC, and Buffer were -0.098 ± 0.021, 0.026 ± 0.022, and -0.012 ± 0.017, respectively **(D)**.

Treatment had a significant effect on changes in the number of eggs per berry (*F*_2,44 =_ 4.89, *p* = 0.012) (**Figures 1 Grape A, B**). GUM treatments resulted in a significant reduction in number of eggs per berry compared to UTC (*t_44_* = -3.05, *p* = 0.011), while no significant difference was found between Buffer and UTC (*t_44_* = -1.42, *p* = 0.339), and between Buffer and GUM (*t_44_* = 1.93, *p* = 0.142). Infestation rates varied significantly among the three treatment levels (*F*_2,44 =_ 5.39, *p* = 0.008) (**Figures 1 Grape C, D**. In particular, the GUM treatment resulted in a significantly reduction in infestation rate compared to UTC (*t_44_* = -3.19, *p* = 0.007), while no significant difference was found between Buffer and UTC (*t_44_* = -1.47, *p* = 0.317), and between Buffer and GUM (*t_44_* = 2.05, *p* = 0.112). The sampling dates and the interactions between sampling date and treatment, and the plot effects were not significant (**Table 1**).

**Table 1:** Statistics generated by using SAS software from trials examining food-grade gum for *D. suzukii* control.

Location	Experiment	Variable Analyzed	Fixed effects			
			Treatment	Sampling Date	Treatment * Sampling Date	Other terms
**Oregon**	**Wine grape**	**Mean number of eggs per berry**	*F*_2,44 =_ 4.89, *p* = 0.012	*F*_2,142_ = 0.08, *p* = 0.920	*F*_4,142_ = 0.68 *p* = 0.608	Plot: *F*_27,44_ = 1.09 *p* = 0.388
	**Wine grape**	**Infestation rate**	*F*_2,44 =_ 5.39, *p* = 0.008	*F*_2,142_ = 0.13, *p* = 0.875	*F*_4,142_ = 0.74 *p* = 0.566	Plot: *F*_27,44_ = 0.92 *p* = 0.579
	**Cherry**	**Infestation rate**	*F*_1,101_ = 1.69 *p* = 0.196	*F*_4,101_ = 176.34 *p* < 0.001	*F*_4,101_ = 0.76 *p* = 0.551	Location: *F*_2,101_ = 8.52 *p* = <0.001
	**Blueberry V1**	**Infestation rate**	$x_{2}^{2}$ =3.08 *p =* 0.215	$x_{1}^{2}$ =33.55 *p <* 0.001	$x_{2}^{2}$ =4.17 *p =* 0.124	Date^2^: $x_{1}^{2}$ =34.71 *p <* 0.001
	**Blueberry V2**	**Infestation rate**	*F*_2,22_ = 4.17 *p* = 0.029	*F*_3,99_ = 14.08 *p* < 0.001	*F*_6,99_ = 1.00 *p* = 0.433	Plot: *F*_11,22_ = 0.68 *p* = 0.744
**California**	**Blueberry V3** **(Screenhouse)**	**Infestation rate**	*F_1,2_* = 52.98 *p* = 0.018	*F_3,12_* = 2.53 *p* = 0.107	*F_3,12_* = 1.94 *p* = 0.177	Plot: *F_2,2_* = 1.09 *p* = 0.478
	**Raspberry V4**	**Mean number of larvae per berry**	*F*_1,5_ = 21.65 *p* = 0.006	*F*_9,90_ = 6.33 *p* < 0.001	*F*_9,90_ = 2.70 *p* = 0.008	Plot: *F_5,5_* = 5.40 *p* = 0.044
	**Raspberry V5, Ranch 1**		*F*_1,5_ = 0.53 *p* = 0.027	*F*_9,90_ = 50.55 *p* < 0.001	*F*_9,90_ = 1.54 *p* = 0.146	Plot: *F_5,5_* = 1.17 *p* = 0.433
	**Raspberry V5, Ranch 2**		*F*_1,5_ = 1.12 *p* = 0.339	*F*_5,50_ = 1.35 *p* = 0.259	*F*_5,50_ = 2.99 *p* = 0.020	Plot: *F_5,5_* = 1.14 *p* = 0.443
	**Raspberry V6**		*F*_1,5_ = 1.82 *p* = 0.235	*F*_5,50_ = 7.99 *p* < 0.001	*F*_5,50_ = 4.20 *p* = 0.003	Plot: *F_5,5_* = 3.11 *p* = 0.119
	**Blackberry V7, Ranch 1**	**Mean number of larvae per berry**	*F*_1,5_ = 2.29 *p* = 0.190	*F*_6,60_ = 14.90 *p* < 0.001	*F*_6,60_ = 3.97 *p* = 0.002	Plot: *F_5,5_* = 3.50 *p* = 0.098
	**Blackberry V7, Ranch 2**		*F*_1,8_ = 0.25 *p* = 0.632	*F*_3,36_ = 15.44 *p* < 0.001	*F*_3,36_ = 3.64 *p* = 0.022	Plot: *F_8,8_* = 0.10 *p* = 0.998
	**Blackberry V7, Ranch 3**		*F*_1,5_ = 19.75 *p* = 0.007	*F*_3,30_ = 35.96 *p* < 0.001	*F*_3,30_ = 0.73 *p* = 0.539	Plot: *F_5,5_* = 3.01 *p* = 0.126
	**Strawberry V8, Ranch 1**	**Mean number of larvae per berry**	*F*_1,5_ = 0.57 *p* = 0.484	*F*_1,10_ = 80.04 *p* < 0.001	*F*_1,10_ = 22.84 *p* < 0.001	Plot: *F_5,5_* = 2.65 *p* = 0.154
	**Strawberry V8, Ranch 2**		*F*_1,5_ = 2.54 *p* = 0.172	*F*_1,10_ = 0.03 *p* = 0.873	*F*_1,10_ = 3.02 *p* = 0.113	Plot: *F_5,5_* = 1.85 *p* = 0.258
	**Strawberry V8, Ranch 3**		*F*_1,5_ = 4.99 *p* = 0.076	*F*_1,10_ = 10.53 *p* = 0.009	*F*_1,10_ = 0.94 *p* = 0.356	Plot: *F_5,5_* = 1.95 *p* = 0.241
	**Blackberry open-field**	**Mean number of eggs per berry**	*F*_2,11_ = 0.15 *p* = 0.859	*F*_8,136_ = 17.09 *p* < 0.001	*F*_16,136_ = 0.77 *p* = 0.716	Plot: *F*_6,11_ = 1.27 *p* = 0.345
	**Blackberry open-field**	**Mean number of larvae per berry**	*F*_2,12_ = 0.20 *p* = 0.823	*F*_9,162_ = 224.98 *p* < 0.001	*F*_18,162_ = 0.89 *p* = 0.594	Plot: *F*_6,12_ = 2.06 *p* = 0.135

#### 5.1.2 Cherry (2019)

**Figure 2:**
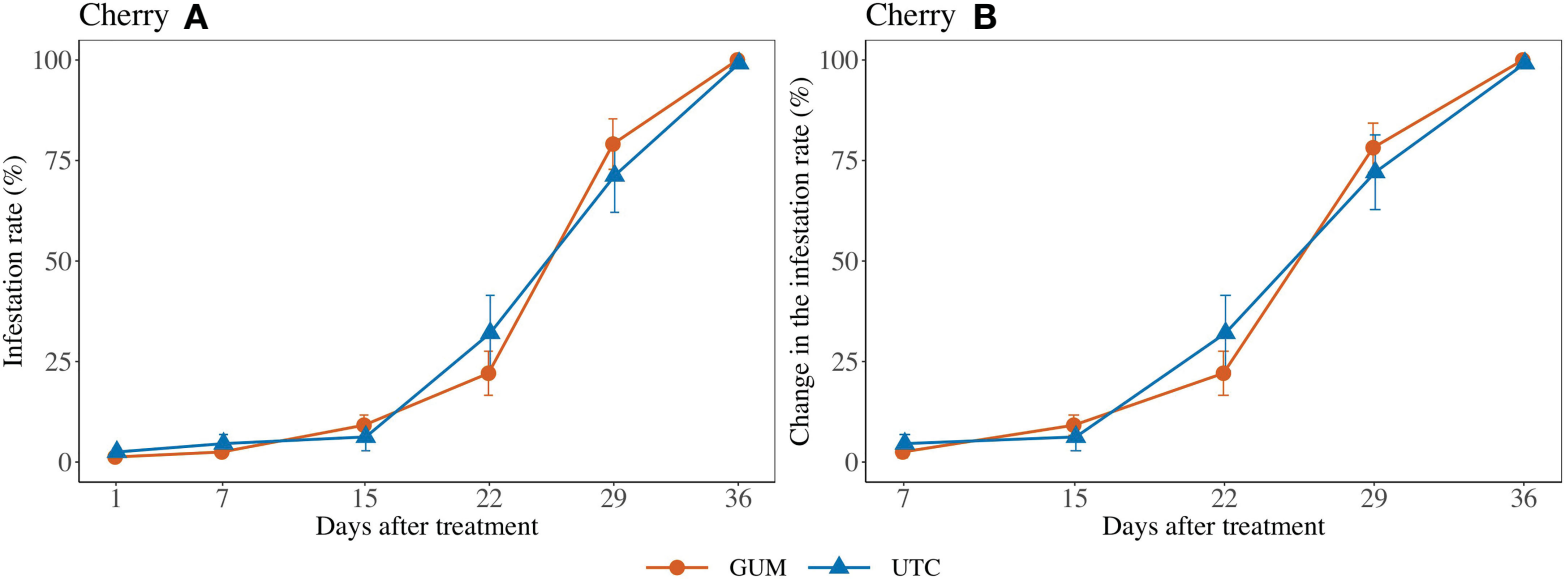
Effect of food-grade gum treatments on *D. suzukii* egg infestation rate (± SEM) on Cherry in Hood River in Oregon during 2020. The sample mean and SE of infestation rates for GUM and UTC were 0.351 ± 0.049 and 0.360 ± 0.049, respectively **(A)**. The sample mean and SE of changes in infestation rate for GUM and UTC were 0.418 ± 0.054 and 0.428 ± 0.055 respectively **(B)**.

No significant difference was found between GUM and UTC in terms of change in infestation rate (*F*_1,101_ = 1.69, *p* = 0.196) (**Figures 2 Cherry A, B**). The sampling date and location had significant effects on change in infestation rate while the treatment and sampling date interaction was not significant (**Table 1**).

#### 5.1.3 Blueberry (2020)

**Figure 3:**
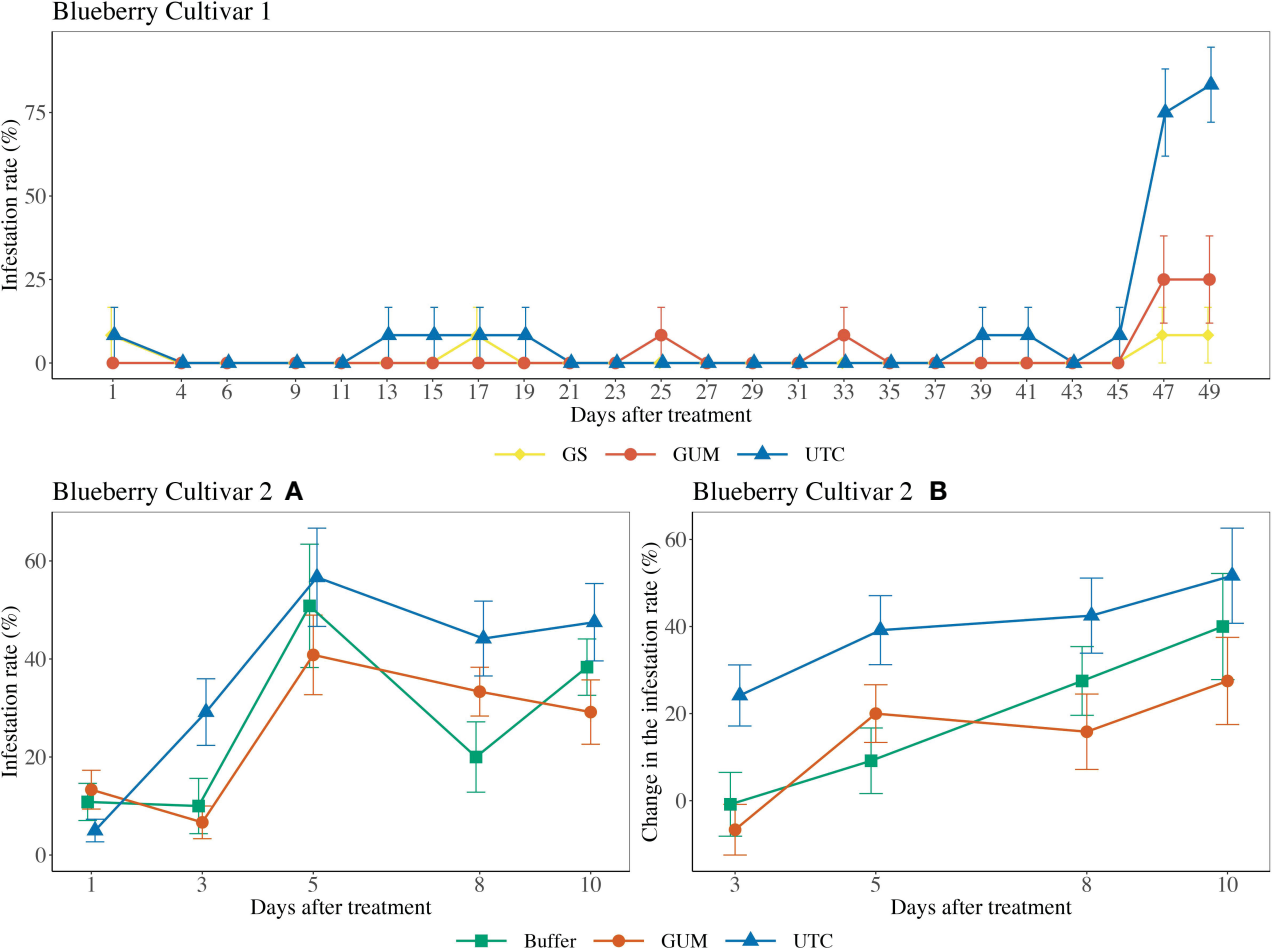
Effect of food-grade gum treatments on *D. suzukii* egg infestation rate (± SEM) on blueberry varieties under commercial conditions in the Willamette Valley, Oregon during 2020. For blueberry cultivar 1, the sample mean and SE of infestation rate for GUM, UTC and GS were 0.028 ± 0.010, 0.094 ± 0.017 and 0.014 ± 0.007 respectively. For blueberry cultivar 2, the sample mean and SE of infestation rate for GUM, UTC and Buffer were 0.247 ± 0.030, 0.365 ± 0.079 and 0.260 ± 0.039 respectively **(A)**. The sample mean and SE of changes in infestation rate for GUM, UTC and Buffer were 0.142 ± 0.043, 0.394 ± 0.045 and 0.190 ± 0.049 respectively **(B)**.

For blueberry cultivar 1, the main effect of treatment was not significant ( $x_{2}^{2}$ = 3.08, *p* = 0.215) (**Figure 3 Blueberry V1**). The sampling and the quadratic of the sampling date were significant, while the treatment and sampling date interaction was not significant (**Table 1**). For blueberry cultivar 2, treatment was found to have a significant effect on changes in the infestation rate (*F*_2,22 =_ 4.17, *p* = 0.029) (**Figure 3 Blueberry V2**). Specifically, the GUM treatment resulted in a significantly lower infestation rate compared with UTC (*t_22_* = -2.66, *p* = 0.037), while no significant difference was found between Buffer and UTC (*t_22_* = -2.03, *p* = 0.076), and between Buffer and GUM (*t_22_* = 0.36, *p* = 0.932). The sampling date had a significant impact on infestation levels, while the interaction between sampling date and treatment and the plot were not significant (**Table 1**).

### 5.2 California

#### 5.2.1 Blueberry screenhouse (2020)

**Figure 4:**
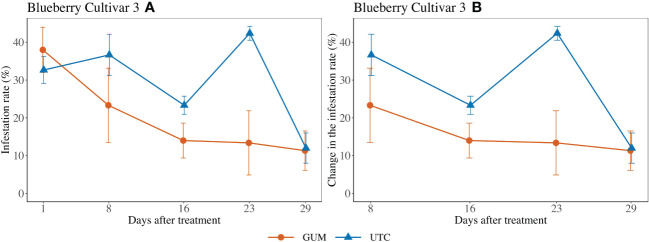
Effect of food-grade gum treatments on *D. suzukii* egg blueberry infestation rate (± SEM) in a screenhouse trial (cultivar 3) in California during 2020. The sample mean and SE of the infestation rate for GUM and UTC were 0.200 ± 0.038 and 0.294 ± 0.0318, respectively **(A)**. The sample mean and SE of changes in infestation rate for GUM and UTC were 0.155 ± 0.034 and 0.286 ± 0.039 **(B)**.

Treatment had a significant impact on infestation rate (*F*_1,2 =_ 52.98, *p* = 0.018) (**Figure 4**). In particular, the GUM plots displayed a reduction in infestation rate compared to UTC plots (*t_2_* = -7.28, *p* = 0.018). Other fixed effects were not significant (**Table 1**).

#### 5.2.2 Raspberry, blackberry, and strawberry (2020)

**Figure 5:**
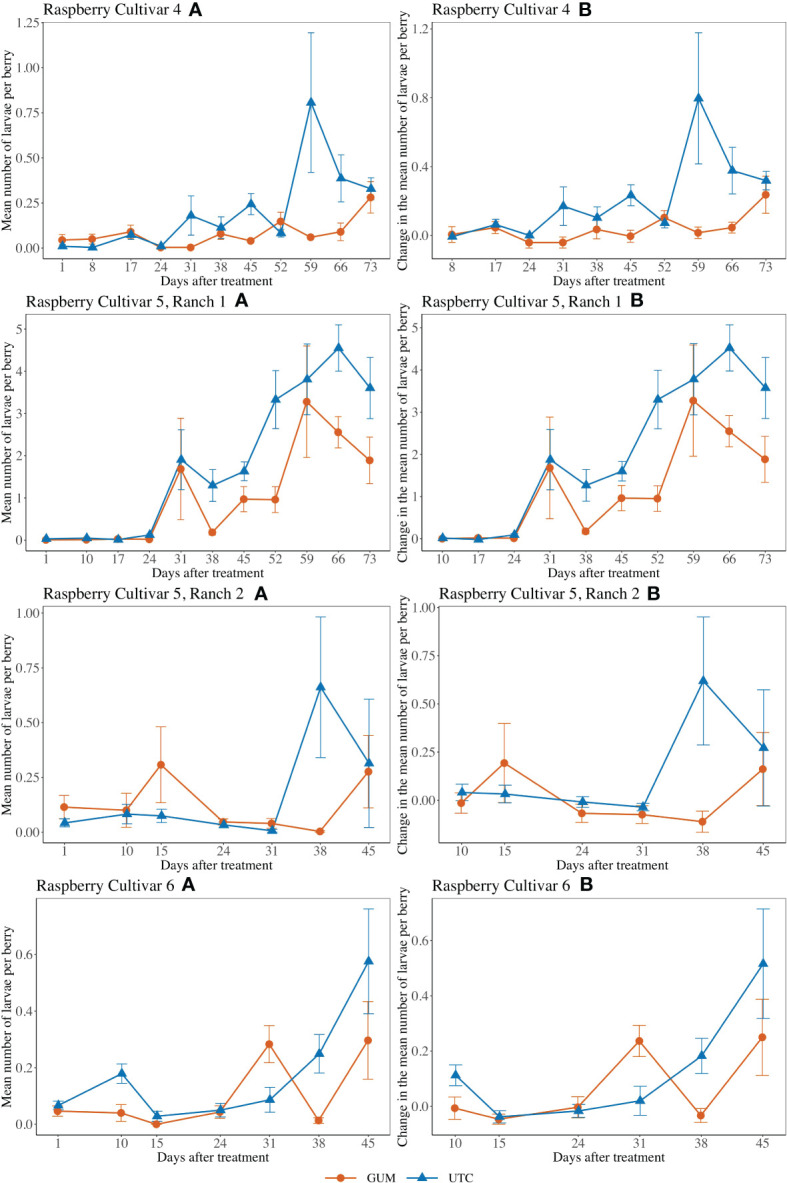
Effect of food-grade gum combined with pesticide (Gum) compared to pesticide only (UTC) treatments on the mean number of D. suzukii larval per berry (± SEM). Experiments were conducted on three commercially produced raspberry varieties under commercial conditions in California during 2020. For raspberry cultivar 4, the sample mean and SE of the number of larvae per berry were 0.081 ± 0.014 and 0.203 ± 0.046 respectively for GUM and UTC (Cultivar 4 **A**). The sample mean and SE of changes in number of larvae per berry for GUM and UTC were 0.040 ± 0.018 and 0.213 ± 0.050 respectively (Cultivar 4 **B**). For cultivar 5 at Ranch 1, the sample mean and SE of the number of larvae per berry for GUM and UTC were 1.054 ± 0.213 and 1.849 ± 0.246 respectively (Ranch 1 **A**). The sample mean and SE of changes in number of larvae per berry for GUM and UTC were 1.152 ± 0.230 and 2.001 ± 0.259 (Ranch 1 **B**). For cultivar 5 at Ranch 2, the sample mean and SE of the number of larvae per berry for GUM and UTC were 0.123 ± 0.038 and 0.174 ± 0.067 respectively (Ranch 2 **A**). The sample mean and SE of changes in number of larvae per berry for GUM and UTC were 0.014 ± 0.050 and 0.153 ± 0.080, respectively (Ranch 2 **B**). For raspberry cultivar 6, the sample mean and SE of the number of larvae per berry for GUM and UTC were 0.103 ± 0.028 and 0.167 ± 0.036 respectively (Cultivar 6 **A**). The sample mean and SE of changes in number of larvae per berry for GUM and UTC were 0.066 ± 0.033 and 0.118 ± 0.043, respectively (Cultivar 6 **B**).

GUM treatments significantly reduced the number of larvae per berry (*F*_1,5 =_ 4.89, *p* = 0.006) (**Figures 5 V4A, B**). Overall, the GUM treatment resulted in a lower increase in number of larvae per berry compared with UTC (*t_5_* = -4.65, *p* = 0.006). However, the main effects of treatment and sampling date were qualified by a significant interaction between treatment and date (*F*_9,90_ = 2.70, *p* = 0.008). The simple effect tests showed that the number of larvae per berry for GUM was significantly lower than UTC at date 31 (*t* = -2.72, *p* = 0.008), date 45 (*t* = -3.24, *p* = 0.002), date 59 (*t* = -4.11, *p* < 0.001) and date 66 (*t* = -3.42, *p* = 0.001), while no significant difference was found at the other dates after Bonferroni adjustment for multiple comparisons. The plot effect was a significant factor (**Table 1**).

For cultivar 5 at Ranch 1, the number of larvae per berry was lower in the GUM compared to the UTC (*F*_1,5_ = 9.53, *p* = 0.027) (**Figures 5 V5R1A, B**). The sampling date was also found to be significant, while plot and the interaction between treatment and date were not significant (**Table 1**). For cultivar 5 at Ranch 2, the main effect of treatment was not significant (*F*_1,5_ = 1.12, *p* = 0.339) (**Figures 5 V5R2A, B**). It was also found that plot and sampling date were not significant (**Table 1**). However, there was an interaction between treatment and date (*F*_5,50_ = 2.99, *p* = 0.020). The simple effect tests suggested that the number of larvae per berry for GUM was lower than UTC on date 38 (*t* = -3.17, *p* = 0.003) after the Bonferroni adjustment for multiple comparisons.

For raspberry cultivar 6, the main effect of treatment was not significant (*F*_1,5_ = 1.82, *p* = 0.235) (**Figures 5 V6A, B**). The interaction between treatment and date (*F*_5,50_ = 4.20, *p* = 0.003) was however significant. The simple effect tests showed that with the Bonferroni adjustment for multiple comparisons, the number of larvae per berry for GUM was significantly higher than UTC at date 31 (*t* = 2.90, *p* = 0.006), and was lower than UTC on date 38 (*t* = -2.92, *p* = 0.005), while no significant difference was found at the other dates. And the sampling date and the plot effect were not significant (**Table 1**).

**Figure 6:**
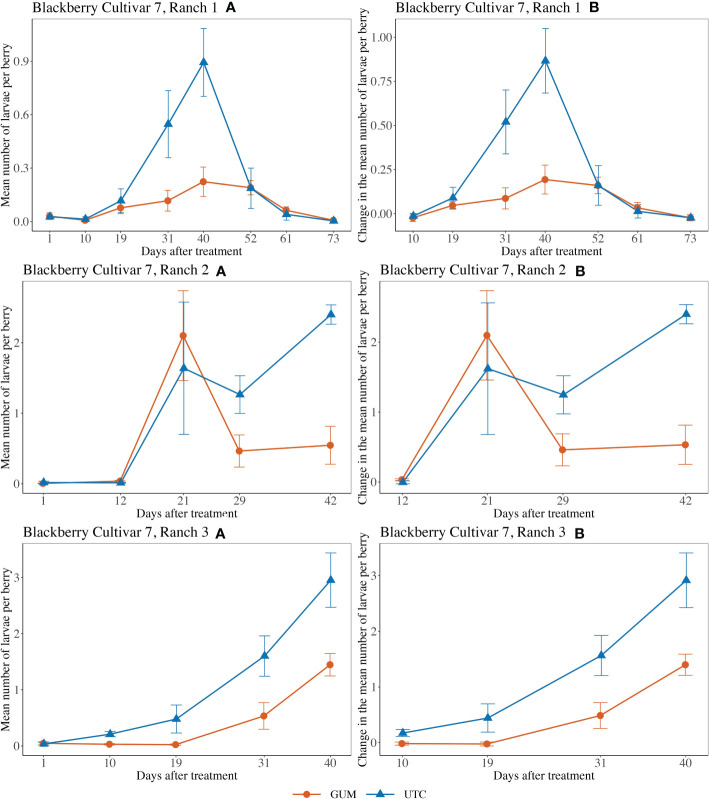
Effect of foodgrade gum combined with pesticide (Gum) compared to pesticide only (UTC) treatments on the mean number of *D. suzukii* larval per berry (± SEM). Experiments were conducted on a commercial blackberry cultivar at three locations in California during 2020. For blackberry cultivar 7 at Ranch 1, the sample mean and SE of the number of larvae per berry for GUM and UTC were 0.089 ± 0.017 and 0.228 ± 0.056 respectively (Ranch 1 **A**). The sample mean and SE of changes in number of larvae per berry for GUM and UTC were 0.068 ± 0.020 and 0.230 ± 0.062, respectively (Ranch 1 **B**). For blackberry cultivar 7 at Ranch 2, the sample mean and SE of the number of larvae per berry for GUM and UTC were 0.644 ± 0.201 and 0.862 ± 0.254 respectively (Ranch 2 **A**). The sample mean and SE of changes in the number of larvae per berry for GUM and UTC were 0.831 ± 0.252 and 1.099 ± 0.318, respectively (Ranch 2 **B**). For blackberry cultivar 7 at Ranch 3, the sample mean and SE of the number of larvae per berry for GUM and UTC were 0.416 ± 0.117 and 1.056 ± 0.237 respectively (Ranch 3 **A**). The sample mean and SE of changes in the number of larvae per berry for GUM and UTC were 0.462 ± 0.140 and 1.275 ± 0.274, respectively (Ranch 3 **B**).

For blackberry cultivar 7 at Ranch 1, the main effect of treatment was not significant (*F*_1,5_ = 2.29, *p* = 0.190) (**Figures 6 Ranch 1A, B**). The interaction between treatment and date was significant (*F*_6,60_ = 3.97, *p* = 0.002). The simple effect tests showed that after the Bonferroni adjustment for multiple comparisons, the number of larvae per berry for GUM was lower than UTC on date 31 (*t* = -3.45, *p* = 0.001 < 0.1/7) and date 40 (*t* = -3.68, *p* = 0.001 < 0.1/7, while no significant difference was found at the other dates). And the sampling data and the plot effect were not significant (**Table 1**). For blackberry cultivar 7 at Ranch 2, the treatment was shown to have no significant effect on the number of larvae per berry (*F*_1,8_ = 0.25, *p* = 0.632) (**Figures 6 Ranch 2A, B**). The interaction between treatment and date was significant (*F*_3,36_ = 3.64, *p* = 0.022). However, the simple effect tests showed that the number of larvae per berry of GUM and UTC did not significantly differ at any date. The sampling date was significant, while the plot effect was not (**Table 1**). For blackberry cultivar 7 at Ranch 3, the treatment had a significant effect on the number of larvae per berry (*F*_1,5_ = 19.75, *p* = 0.007) (**Figures 6 Ranch 3A, B**). The sampling date was a significant factor while the treatment and date interaction and the plot effect were not (**Table 1**).

**Figure 7:**
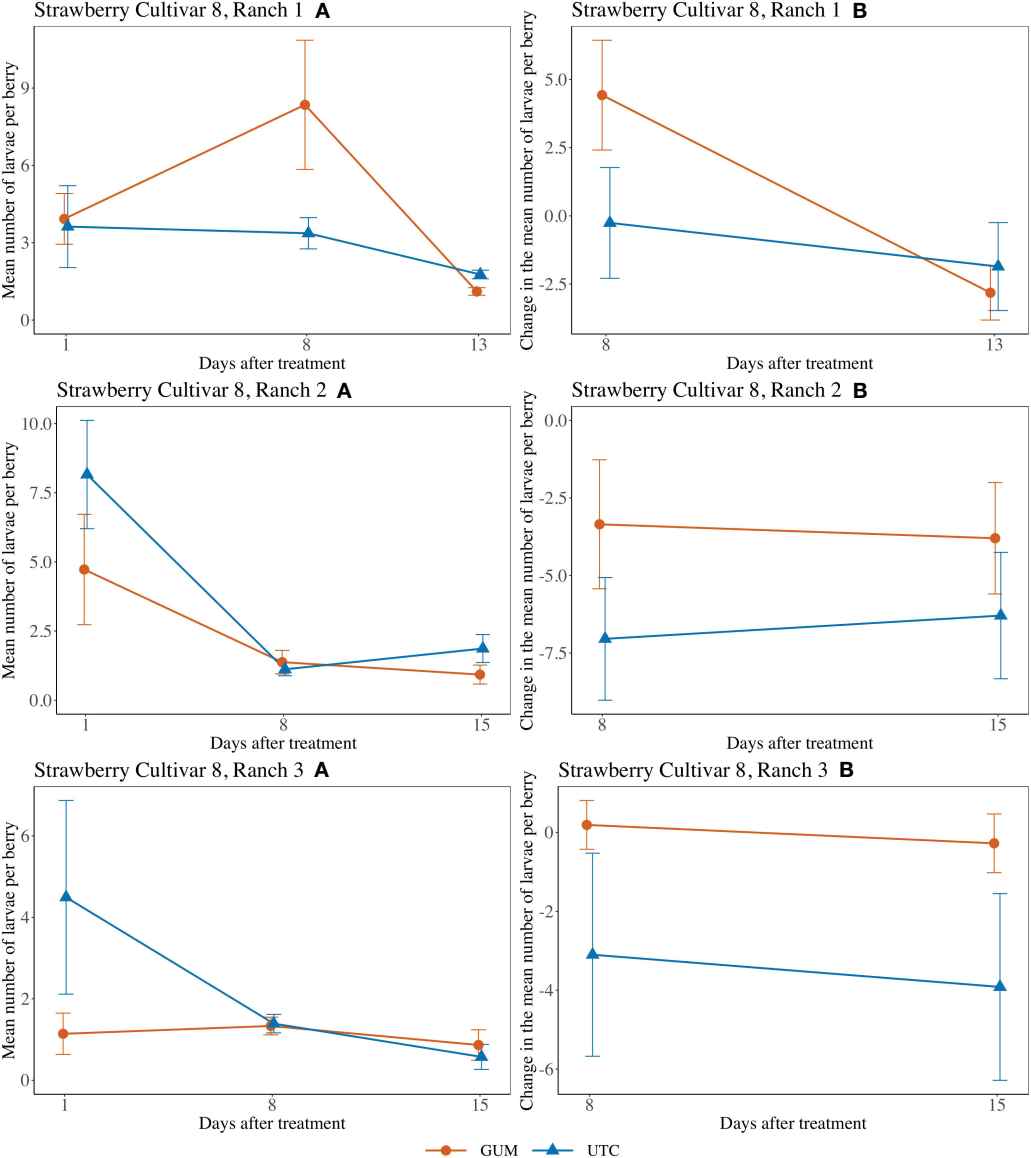
Effect of foodgrade gum combined with pesticide (Gum) compared to pesticide only (UTC) treatments on the mean number of *D. suzukii* larval per berry (± SEM). Experiments were conducted on a commercial strawberry cultivar at three locations in California during 2020. For strawberry cultivar 8 at Ranch 1, the sample mean and SE of the number of larvae per berry for GUM and UTC were 4.461 ± 1.111 and 2.919 ± 0.571 respectively (Ranch 1 **A**). The sample mean and SE of changes in the number of larvae per berry for GUM and UTC were 0.804 ± 1.530 and -1.058 ± 1.259, respectively (Ranch 1 **B**). For strawberry cultivar 8 at Ranch 2, the sample mean and SE of the number of larvae per berry for GUM and UTC were 2.342 ± 0.767 and 3.714 ± 0.996 respectively (Ranch 2 **A**). The sample mean and SE of changes in the number of larvae per berry for GUM and UTC were -3.575 ± 1.311 and -6.667 ± 1.358, respectively (Ranch 2 **B**). For strawberry cultivar 8 at Ranch 3, the sample mean and SE of the number of larvae per berry for GUM and UTC were 1.114 ± 0.213 and 2.153 ± 0.858 respectively (Ranch 3 **A**). The sample mean and SE of changes in the number of larvae per berry for GUM and UTC were -0.042 ± 0.467 and -3.508 ± 1.672, respectively (Ranch 3 **B**).

For strawberry cultivar 8 at Ranch 1, the treatment had no significant effect on the number of larvae per berry (*F*_1,5_ = 0.57, *p* = 0.484) (**Figures 7 Ranch 1 A, B**). The interaction between treatment and date (*F*_1,10_ = 22.84, *p* < 0.001) was significant. However, the simple effect tests showed that with the Bonferroni adjustment for multiple comparisons, the numbers of larvae per berry for GUM and UTC were not significantly different at any date. The sampling date was significant while the plot effect was not (**Table 1**). For strawberry cultivar 8 at Ranch 2, it was found that none of the fixed effects had a significant effect on the number of larvae per berry (**Figures 7 Ranch 2 A, B**) (**Table 1**). For strawberry cultivar 8 at Ranch 3, the number of larvae was significantly higher in GUM than UTC (*F*_1,5_ = 4.99, *p* = 0.076) (**Figures 7 Ranch 3 A, B**). The sampling date was significant while the interaction between treatment and date interaction and the plot effect were not (**Table 1**).

#### 5.2.3 Blackberry (2020)

**Figure 8:**
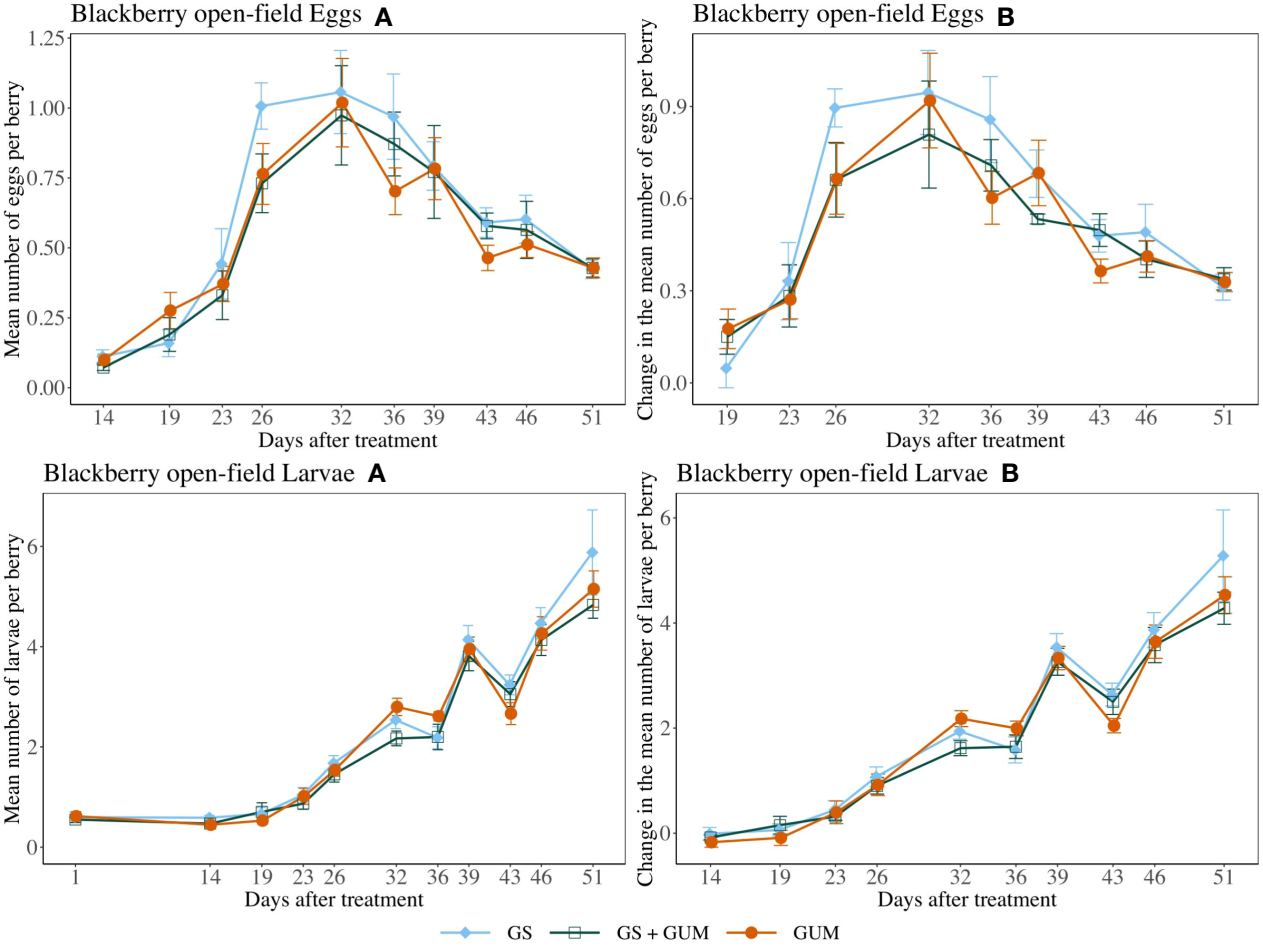
Effect of food-grade gum as standalone (GUM), in combination with a pesticide (GS+GUM) compared to pesticide only (UTC) treatments on the mean number of *D. suzukii* egg and larvae per berry (± SEM). Experiments were conducted on a commercial strawberry cultivar at three locations in California during 2020. For egg counts, the sample mean and SE of the number of eggs per berry for GUM, GS and GUM+GS were 0.542 ± 0.040, 0.616 ± 0.048 and 0.558 ± 0.046 respectively (Eggs **A**). The sample mean and SE of changes in the number of eggs per berry for GUM, GS and GUM+GS were 0.491 ± 0.040, 0.560 ± 0.047 and 0.487 ± 0.039 respectively (Eggs **B**). For larvae counts, the sample mean and SE of the number of larvae per berry for GUM, GS and GUM+GS were 2.327 ± 0.188, 2.456 ± 0.215 and 2.205 ± 0.180 respectively (Larvae **A**). The sample mean and SE of changes in the number of larvae per berry for GUM, GS and GUM+GS were 1.879 ± 0.195, 2.041 ± 0.226 and 1.818 ± 0.187 respectively (Larvae **B**).

For both egg counts and larvae counts, there was no significant difference in treatments in terms of change in the number of eggs or larvae per berry (egg counts*: F*_2,11_ = 0.15, *p* = 0.859, larvae count: *F*_2,12_ = 0.20, *p* = 0.823) (**Figure 8**). The sampling dates were significant, while the treatment and sampling date interaction and the plot effect were not significant (**Table 1**).

### 5.3 Joint test of GUM vs UTC

Meta-analysis to determine difference between GUM and UTC and mean larvae resulted in a highly significant p-value using Stouffer’s method. It suggested that the joint null hypothesis of no difference between GUM and UTC can be rejected (Z=3.476, p<0.001).

## 6 Population modeling

**Figure 9:**
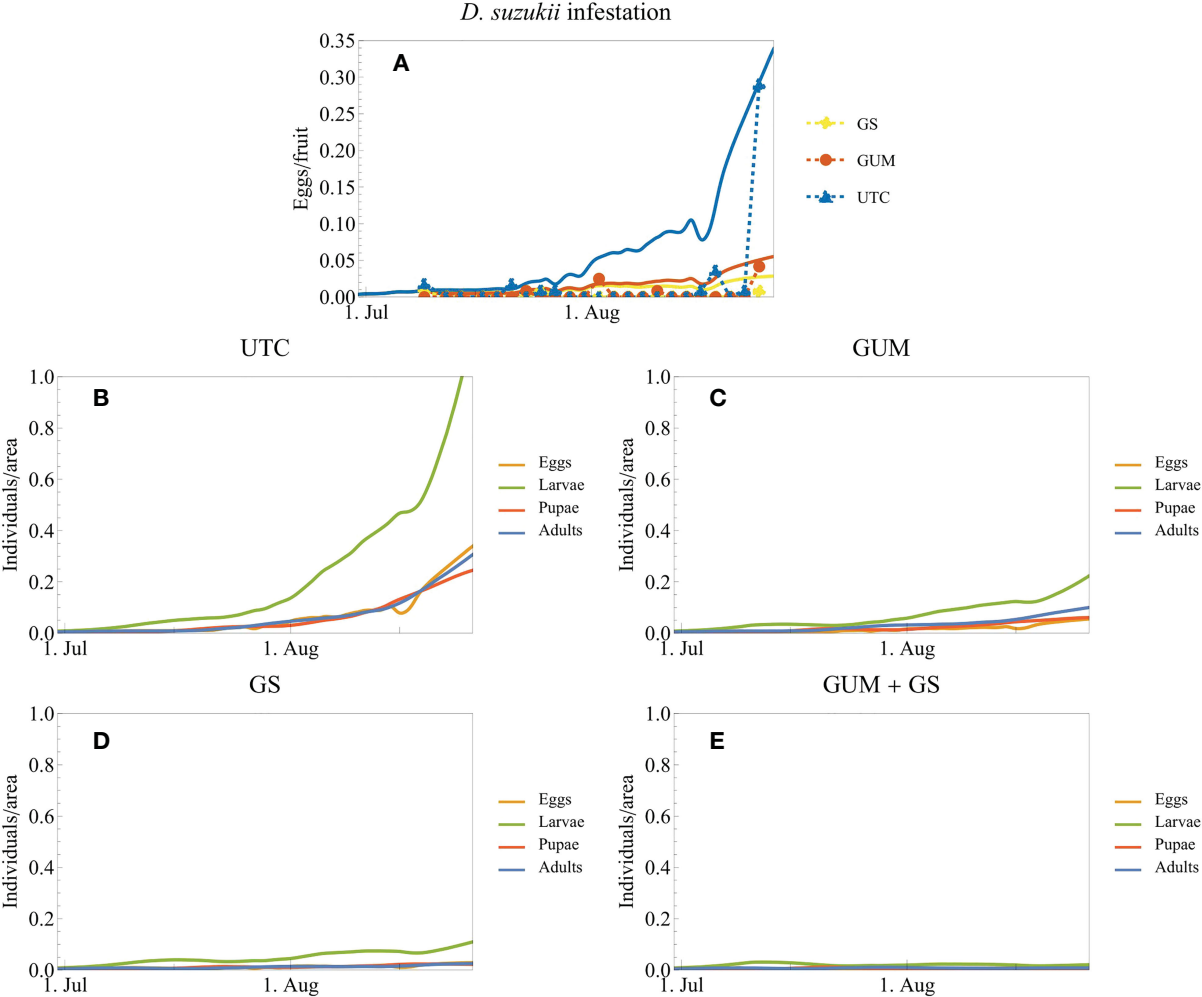
Simulated *D. suzukii* population dynamics and infestation data from trial 1 (blueberry open-field experiment in Oregon 2020). The field experiments and the corresponding simulations **(A)** cover three treatments: untreated control (UTC), gum application without insecticides (GUM), and insecticide application (growers standard, GS). The plot markers and dashed lines correspond to the experimental data, and the solid lines correspond to model simulations. It was assumed that the number of eggs/fruits is proportional to the density of eggs in the field. **(B-E)** additionally show the densities of the other life stages of the population. **(E)** shows simulations of a combined treatment of GUM and GS; this treatment was not part of this experimental trial.

The simulations of *D. suzukii* population dynamics demonstrated the buildup of pest populations for each of three treatments. The simulations fit the field data well (**Figure 9A**). Without intervention, the infestation level increased dramatically by the end of the trial (**Figures 9A, B**). Both GUM and GS (insecticide) reduced the infestation level drastically (**Figures 9C, D**). Model outputs suggest that GUM used together with insecticides resulted in further/additional reduction in *D. suzukii* population levels, compared to where pesticides were used as a standalone treatment (**Figures 9C–E**). The final *D. suzukii* population adding all life stages was reduced by the following percentages relative to UTC: UTC 0%, GUM 79%, GS 91%, GUM + GS 98%.

## 7 Discussion

The current study supports findings from previous laboratory and small-scale field cage trials. Here we show through field-collected and modeled data that food-grade gum use can reduce *D. suzukii* fruit damage (28, 29). The aim of this work was to acquire detailed knowledge about limitations of food-grade gum in a range of commercial cropping systems including blueberry, blackberry, cherry, raspberry, strawberry, and winegrape. These studies were conducted in two key production regions i.e., California and Oregon in the USA. The overall results supported initial findings (28, 29) and provided additional evidence that this tool can reduce *D. suzukii* crop damage especially when applied together with the grower standard. Both field-collected data and model simulations indicates that there is a synergistic effect of food-grade gum when used in combination with a conventional insecticide.

For most of the experiments (see **Figures 1**, **3**–**6**), field plots receiving the food-grade gum resulted in either numerical or statistical differences in *D. suzukii* damage compared to untreated control plots. This was not recorded for the cherry, strawberry, and blackberry (Davis) trials. Reasonable hypothesis about these data are discussed below. In trials where *D. suzukii* infestations were measured in buffer plots (**Figures 1**, **3** cultivar 2), there was evidence of a reduction in damage, but not at the same level as in plots treated by the food-grade gum.

Overall, considering all the trials, crop damage was reduced up to 78% (low of 0%) over a period of up to 21 days post application of the food-grade gum. The results from the current study indicate that the food-grade gum can be used in combination with standard insecticides (commercial small fruit production), and in some cases as a stand-alone treatment (winegrape) to reduce the infestation level of *D. suzukii*. Similar reductions in *D. suzukii* damage were reported under laboratory (29) and controlled semi-field conditions (28), suggesting that the food-grade gum resulted in lower damage due to oviposition. These findings support earlier results where the effects of semiochemical volatiles emanating from the food-grade gum resulted in significant behavioral changes (39). In several trials, data lower oviposition and fruit infestation in the presence of the food-grade gum under field conditions. Reasons of why in multiple trials a statistical difference was not reached, can be explained by multiple parameters observed by scientists and growers such as animals removing the cottons pads, water-irrigation issues, and wind. These factors are addressed in a future publication (Tait et al., 2023 in preparation).

In the Hood River cherry trial, constant windy conditions may have resulted in dispersion of volatiles, ultimately resulting in impacts that were less pronounced. There is little doubt that efficiency of the food-grade gum can vary depending on production conditions and crop (28, 29). Host preference of *D. suzukii* was ranked 4^th^ for cherry, followed by blueberry (6^th^) and winegrape (7^th^) (40). Such differences in host preference should be considered when applying food-grade gum. Synthetic blends can be less attractive compared to the actual fruit; thus, additional adjustments may be required to minimize egg-laying in the fruit. Results showed that the application of the food-grade gum in grape shows clear impacts to protect berries from *D. suzukii* attack. Considering the vulnerability of several winegrape cultivars towards *D. suzukii* (41–43) and the encouraging results collected, we have reasons to believe that the food-grade gum can be a useful tool for the winegrape production. For the food-grade gum applications in blueberry in open field experiments, the infestation rate for the food-grade gum and grower standard were 70% and 85% lower than that for untreated control respectively, with the food-grade gum treatment resulted in a significantly lower infestation rate compared with the control. Open and semi-field experiments conducted in California provide similar outcomes to those in Oregon. Blueberry experiments conducted in California within a screenhouse provided 45.5% egg reduction. There were sequential applications with differing timing (weekly starting in April until May) and the results indicated that early applications resulted in lower egg reductions (40.82-79.23%). A potential hypothesis for this phenomenon could be related to environmental conditions including temperature and humidity that could significantly change the emission of plant volatiles (44). Egg reduction in raspberry and blackberry varied from 42-90% and 24-70% respectively. Two cultivars of raspberry have been subjected to the trial and in both cases there was reduction in egg infestation. For blackberry the same cultivar has been evaluated but in three different farms. Results were consistent between the different locations. For strawberry, in several cases results showed numerically increased larval levels compared in the food-grade gum treatments. A potential hypothesis for this phenomenon could be related to either unreported production practices or environmental conditions that could significantly change the emission of plant volatiles or the food-grade gum. Other reasons that can justify the negative results, range from lack of irrigation to rodents removing food-grade gum within a day of placement (growers in person observations). The trial run in Watsonville, California, showed a numerical reduction of eggs when the food-grade gum was applied as standalone and in combination with pesticide. As discussed previously, multiple factors may have impacted the trial. Meta-analysis to determine differences between food-grade gum and untreated control and mean larvae resulted in a highly significant difference. Despite the non-statistical significant results gotten in multiple trials, the meta-analysis showed that by analyzing together all the trials, the food-grade gum has a significant positive effect on protecting fruits from *D. suzukii* infestation. The data originated clearly indicate that the presence of the food-grade gum substrate is a valid approach to keep *D. suzukii* away from berries. This analysis seems extremely valuable because it provides a general idea of how, overall, the use of the new tool has the potential to bring benefits to the small berry industries all over the world.

The parameters used for modeling simulations were obtained from previous laboratory and semi-field experiments. For this study, the initial *D. suzukii* adult densities were fitted to match the untreated control treatment. The relatively similar trends displayed between simulations and real data suggest that model assumptions are close to representative of treatments. Simulation outputs however differed slightly from the field data in the earlier phase of the season. The simulations suggest an earlier buildup of *D. suzukii* populations compared to the sudden increase of infestation in the field trial. A reason for this difference could be that the model output was compared with the experimental data by assuming that the simulated egg population is proportional to the mean number of eggs found per fruit in the experiments. This assumption is reasonable for constant fruit levels, but the availability of ripe fruit in the trials were not constant. Under commercial field conditions, fruit is harvested every 7-10 days for this cultivar. This means that less susceptible fruit is available directly after every harvest event, likely negatively impacting *D. suzukii* population levels. Therefore, a high availability of ripe fruit in the middle portion of the experiment likely resulted in fewer eggs laid per berry compared to later in the season when fruit are less available. These differences in ovipositional resources likely resulted in the sudden increase in recorded infestation levels towards the latter portion of the experiment. Future work should focus on these relationships of pest population level and crop availability to determine risk.

Finally, data collected under different environmental conditions over periods ranging from 10 to 60 days do not appear to impact the efficacy of the food-grade gum. Treated fruits were less damaged by *D. suzukii*. Additional factors such as active distance, commercial field longevity and improved formulation will result in additional improvements and future adoption.

## 8 Conclusion

GUM was an effective non-chemical tool that was used successfully to manage D. suzukii in commercial production fields of small fruit, tree fruit and wine grapes.

As demonstrated in Oregon, the technology can works as a standalone for short or prolonged periods, under various levels of field pressure, cropping systems and environmental conditions. California data displayed a synergistic effect when used in combination with conventional insecticides. Overall, these data suggest the value of this technique to manage *D. suzukii* within a holistic IPM production system. These findings suggest that crop quality would be improved if growers included this technology as part of their management programs.

## Data availability statement

The raw data supporting the conclusions of this article will be made available by the authors, without undue reservation.

## Author contributions

Conceptualization: GT and VW. Methodology: VW, GT, JK, FG, FZ, FP and CA. Formal analysis and investigation: GT, TZ, LX and FP. Field work: GT, JK, FG, RK, HT, CA, EM, FZ and VW. Writing- original draft preparation: GT. Writing – review and editing: CC, FP, SM, FG, FZ, TZ, LX, MS and VW. Funding acquisition: VW and FZ. Supervision: VW. All authors contributed to the article and approved the submitted version.

## Footnotes

1
https://github.com/ferdi-p/gum-simulation-2022.

## References

[1] Hulme PE. Trade, transport and trouble: managing invasive species pathways in an era of globalization. JJ Appl Eco (2009) 46(1):10–8. doi: 10.1111/j.1365-2664.2008.01600.x

[2] Early R Bradley BA Dukes JS Lawler JJ Olden JD Blumenthal DM. Global threats from invasive alien species in the twenty-first century and national response capacities. Nat Commun (2016) 7(1):12485. doi: 10.1038/ncomms12485 27549569 PMC4996970

[3] Gurevitch J Padilla DK. Are invasive species a major cause of extinctions? Trends Ecol Evol (2004) 19(9):470–4. doi: 10.1016/j.tree.2004.07.005 16701309

[4] Venette RC Hutchison WD. Invasive insect species: Global challenges, strategies & opportunities. Front Insect Science. (2021) 1:650520. doi: 10.3389/finsc.2021.650520 PMC10926476 38468878

[5] Panini M Manicardi GC Moores GD Mazzoni E. An overview of the main pathways of metabolic resistance in insects. ISJ (2016) 13(1):326–35. doi: 10.25431/1824-307X/isj.v13i1.326-335

[6] Meyerson LA Mooney HA. Invasive alien species in an era of globalization. F Front Ecol Environ (2007) 5(4):199–208. doi: 10.1890/1540-9295(2007)5[199:IASIAE]2.0.CO;2

[7] Tait G Vezzulli S Sassù F Antonini G Biondi A Baser N. Genetic variability in Italian populations of *Drosophila suzukii* . BMC Genet (2017) 18(1):87. doi: 10.1186/s12863-017-0558-7 29096606 PMC5669006

[8] Heimpel GE Yang Y Hill JD Ragsdale DW. Environmental consequences of invasive species: greenhouse gas emissions of insecticide use and the role of biological control in reducing emissions. PLoS One (2013) 8(8):e72293. doi: 10.1371/journal.pone.0072293 23977273 PMC3748099

[9] Fraimout A Loiseau A Price DK Xuéreb A Martin JF Vitalis R. New set of microsatellite markers for the spotted-wing *Drosophila suzukii* (Diptera: Drosophilidae): a promising molecular tool for inferring the invasion history of this major insect pest. Eur J Entomol (2015) 112:855. doi: 10.14411/eje.2015.079

[10] Hauser M. A historic account of the invasion of *Drosophila suzukii* (Matsumura) (Diptera: Drosophilidae) in the continental united states, with remarks on their identification. Pest Manag Sci (2011) 67(11):1352–7. doi: 10.1002/ps.2265 21898759

[11] Walsh DB Bolda MP Goodhue RE Dreves AJ Lee J Bruck DJ. *Drosophila suzukii* (Diptera: Drosophilidae): Invasive pest of ripening soft fruit expanding its geographic range and damage potential. J Integr Pest Manage (2011) 2(1):G1–7. doi: 10.1603/IPM10010

[12] Deprá M Poppe JL Schmitz HJ De Toni DC Valente VLS. The first records of the invasive pest *Drosophila suzukii* in the south American continent. J Pest Sci (2014) 87(3):379–83. doi: 10.1007/s10340-014-0591-5

[13] Andreazza F Bernardi D Dos Santos RSS Garcia FRM Oliveira EE Botton M. Drosophila suzukii in southern Neotropical region: Current status and future perspectives. Neotrop Entomol (2017) 46(6):591–605. doi: 10.1007/s13744-017-0554-7 28852987

[14] Hassani IM Behrman EL Prigent SR Gidaszewski N Ravaomanarivo LHR Suwalski A. First occurrence of the pest *Drosophila suzukii* (Diptera: Drosophilidae) in the Comoros archipelago (Western Indian ocean). Afr Entomol (2020) 28(1):78–83. doi: 10.4001/003.028.0078

[15] Ometto L Cestaro A Ramasamy S Grassi A Revadi S Siozios S. Linking genomics and ecology to investigate the complex evolution of an invasive drosophila pest. Genome Biol Evol (2013) 5(4):745–57. doi: 10.1093/gbe/evt034 PMC3641628 23501831

[16] Goodhue RE Bolda M Farnsworth D Williams JC Zalom FG. Spotted wing drosophila infestation of California strawberries and raspberries: economic analysis of potential revenue losses and control costs. Pest Manag Sci (2011) 67(11):1396–402. doi: 10.1002/ps.2259 21815244

[17] Yeh DA Drummond FA Gómez MI Fan X. The economic impacts and management of spotted wing drosophila (*Drosophila suzukii*): The case of wild blueberries in Maine. J J Econ Entomol (2020) 113(3):1262–9. doi: 10.1093/jee/toz360 PMC7275691 31943106

[18] Tait G Mermer S Stockton D Lee J Avosani S Abrieux A. Drosophila suzukii (Diptera: Drosophilidae): A decade of research towards a sustainable integrated pest management program. J Econ Entomol (2021) 114(5):1950–74. doi: 10.1093/jee/toab158 34516634

[19] Knapp L Mazzi D Finger R. The economic impact of *Drosophila suzukii*: perceived costs and revenue losses of Swiss cherry, plum and grape growers. Pest Manag Scie. (2021) 77(2):978–1000. doi: 10.1002/ps.6110 PMC7821377 32990345

[20] Bolda MP Goodhue R Zalom F. Spotted wing drosophila: potential economic impact of a newly established pest. Giannini Foundation Agric Econ (2010) 13:5–8.

[21] Mermer S Pfab F Tait G Isaacs R Fanning PD Van Timmeren S. Timing and order of different insecticide classes drive control of *Drosophila suzukii*; a modeling approach. J Pest Sci (2021) 94(3):743–55. doi: 10.1007/s10340-020-01292-w

[22] Krüger AP Scheunemann T Padilha AC Pazini JB Bernardi D Grützmacher AD. Insecticide-mediated effects on mating success and reproductive output of *Drosophila suzukii* . Ecotoxicology (2021) 30(5):828–35. doi: 10.1007/s10646-021-02402-9 33851336

[23] Civolani S Vaccari G Caruso S Finetti L Bernacchia G Chicca M. Evaluation of insecticide efficacy and insecticide adaptive response in Italian populations of drosophila suzukii. Bull Insectol (2021) 74:103–14.

[24] Jepson PC Murray K Bach O Bonilla MA Neumeister L. Selection of pesticides to reduce human and environmental health risks: a global guideline and minimum pesticides list. Lancet Planet Health (2020) 4(2):e56–63. doi: 10.1016/S2542-5196(19)30266-9 32112748

[25] Gress BE Zalom FG. Identification and risk assessment of spinosad resistance in a California population of *Drosophila suzuki*i. Pest Manag Scie. (2019) 75(5):1270–6. doi: 10.1002/ps.5240 30324771

[26] Ganjisaffar F Gress BE Demkovich MR Nicola NL Chiu JC Zalom FG. Spatio-temporal variation of spinosad susceptibility in *Drosophila suzukii* (Diptera: Drosophilidae), a three-year study in california’s Monterey bay region. J Econ Entomol (2022) 115(4):972–80. doi: 10.1093/jee/toac011 35137165

[27] Schöneberg T Lewis MT Burrack HJ Grieshop M Isaacs R Rendon D. Cultural control of *Drosophila suzukii* in small fruit–current and pending tactics in the US. Insects (2021) 12(2):172. doi: 10.3390/insects12020172 33671153 PMC7923098

[28] Rossi Stacconi MV Tait G Rendon D Grassi A Boyer G Nieri R. Gumming up the works: Field tests of a new food-grade gum as behavioral disruptor for *Drosophila suzukii* (Diptera: Drosophilidae). J Econ Entomol (2020) 113(4):1872–80. doi: 10.1093/jee/toaa072 32333602

[29] Tait G Kaiser C Stacconi R Dalton DT Anfora G Walton VM. A food-grade gum as a management tool for *Drosophila suzukii* . Bull Insect. (2018) 71(2):295–307.

[30] Dalton DT Walton VM Shearer PW Walsh DB Caprile J Isaacs R. Laboratory survival of *Drosophila suzukii* under simulated winter conditions of the pacific Northwest and seasonal field trapping in five primary regions of small and stone fruit production in the united states. Pest Manag Sci (2011) 67(11):1368–74. doi: 10.1002/ps.2280 22021034

[31] Van Timmeren S Sial AA Lanka SK Spaulding NR Isaacs R. Development of a rapid assessment method for detecting insecticide resistance in spotted wing drosophila (*Drosophila suzukii* matsumura). Pest Manag Sci (2019) 75(7):1782–93. doi: 10.1002/ps.5341 30653815

[32] Firth D. Bias reduction of maximum likelihood estimates. Biometrika (1993) 80(1):27–38. doi: 10.1093/biomet/80.1.27

[33] Heard N Rubin-Delanchy P. Choosing between methods of combining p-values. Biometrika (2018) 105(1):239–46. doi: 10.1093/biomet/asx076

[34] Tochen S Dalton DT Wiman N Hamm C Shearer PW Walton VM. Temperature-related development and population parameters for *Drosophila suzukii* (Diptera: Drosophilidae) on cherry and blueberry. Environ Entomol (2014) 43(2):501–10. doi: 10.1603/EN13200 24612968

[35] Emiljanowicz LM Ryan GD Langille A Newman J. Development, reproductive output and population growth of the fruit fly pest *Drosophila suzukii* (Diptera: Drosophilidae) on artificial diet. J Econ Entomol (2014) 107(4):1392–8. doi: 10.1603/EC13504 25195427

[36] Pfab F Stacconi MVR Anfora G Grassi A Walton V Pugliese A. Optimized timing of parasitoid release: a mathematical model for biological control of *Drosophila suzukii* . Theor Ecol (2018) 11(4):489–501. doi: 10.1007/s12080-018-0382-3

[37] Mermer S Pfab F Hoheisel GA Bahlol HY Khot L Dalton DT. Canopy spray deposition and related mortality impacts of commonly used insecticides on *Drosophila suzukii* matsumura (Diptera: Drosophilidae) populations in blueberry. Pest Manag Scie. (2020) 76(4):1531–40. doi: 10.1002/ps.5672 31692223

[38] Latest features in Mathematica 13. Available at: https://www.wolfram.com/mathematica/new-in-13/.

[39] Tait G Park K Nieri R Crava MC Mermer S Clappa E. Reproductive site selection: Evidence of an oviposition cue in a highly adaptive dipteran, *Drosophila suzukii* (Diptera: Drosophilidae). Environ Entomol (2020) 49(2):355–63. doi: 10.1093/ee/nvaa005 31977012

[40] Abraham J Zhang A Angeli S Abubeker S Michel C Feng Y. Behavioral and antennal responses of *Drosophila suzukii* (Diptera: Drosophilidae) to volatiles from fruit extracts. Environ Entomo. (2015) 44(2):356–67. doi: 10.1093/ee/nvv013 26313190

[41] Ioriatti C Walton V Dalton D Anfora G Grassi A Maistri S. *Drosophila suzukii* (Diptera: Drosophilidae) and its potential impact to wine grapes during harvest in two cool climate wine grape production regions. J Econ Entomol (2015) 108(3):1148–55. doi: 10.1093/jee/tov042 26470240

[42] Baser N Broutou O Verrastro V Porcelli F Ioriatti C Anfora G. Susceptibility of table grape varieties grown in south-eastern Italy to drosophila suzukii. J Appl Entomol (2018) 142(5):465–72. doi: 10.1111/jen.12490

[43] Entling W Anslinger S Jarausch B Michl G Hoffmann C. Berry skin resistance explains oviposition preferences of *Drosophila suzukii* at the level of grape cultivars and single berries. J Pest Sci (2019) 92(2):477–84. doi: 10.1007/s10340-018-1040-7

[44] Vallat A Gu H Dorn S. How rainfall, relative humidity and temperature influence volatile emissions from apple trees in situ. Phytochemistry (2005) 66(13):1540–50. doi: 10.1016/j.phytochem.2005.04.038 15949824

