# Supplementary material for: Open field trials of food-grade gum in California and Oregon as a behavioral control for Drosophila suzukii Matsumura (Diptera: Drosophilidae)
Source: Front Insect Sci. 2023 May 2;3:1141853. doi: 10.3389/finsc.2023.1141853 (PMC10926501; doi:10.3389/finsc.2023.1141853)
Supplement: Supplementary file 1 [file DataSheet_1.docx]

**Supplementary Material**

**Title:** Open field trials of food-grade gum in California and Oregon as a behavioral control for *Drosophila suzukii* Matsumura (Diptera: Drosophilidae)

**Authors**: Gabriella Tait, Tingyu Zhu, Jimmy Klick, Fatemeh Ganjisaffar, Claira Castillo, Ryan Kennedy, Hillary Thomas, Christopher Adams, Ferdinand Pfab, Serhan Mermer, Enrico Mirandola, Lan Xue, Frank G. Zalom, Michael Seagraves, Vaughn M. Walton

**Contact for Supplementary Material**: ferdinand.pfab@gmail.com

**Model**

This document describes the model used in the main text to simulate the population dynamics of *D. suzukii* under different schemes for insecticide applications. The model implements a stage structured approach with eggs E, larvae L, pupae P and adults A. The simulations are driven by temperature. Variants of this model have been previously used in (1,2). Parts of the model description and some of the figures here are reproductions from these earlier papers.

Model equations

Data for fecundity, maturation delays, adult life length and juvenile mortality were taken from (3), who measured these traits with individuals reared on blueberry at different temperatures, see

Figure 1. Temperature dependent properties. In this source, the egg and larva durations were measured together. To render our model more general, we split the stage in eggs and larvae using the proportions from (4).

When not mentioned otherwise we use the data measured on female individuals. For the functional fits we used Gaussian-like functions

$$f\left( c,\mu,\sigma;w \right)=c e^{-\left( \frac{\mu-w}{\sigma} \right)^{2}}$$

and

$$g\left( c,\mu,\sigma;w \right)=c e^{+\left( \frac{\mu-w}{\sigma} \right)^{2}}$$

Where w is the current temperature, $\mu$ is the optimal temperature, $\sigma$ is the breadth of tolerance, and$c$ is the maximal or minimal value for $f$ and $g$ respectively.

| 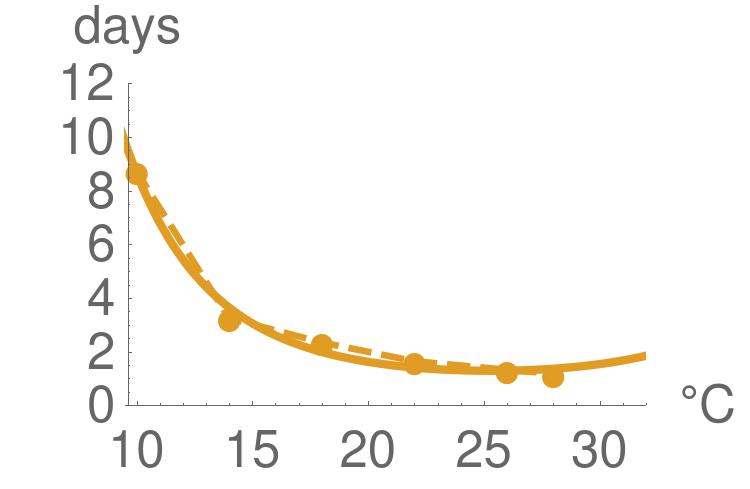 | 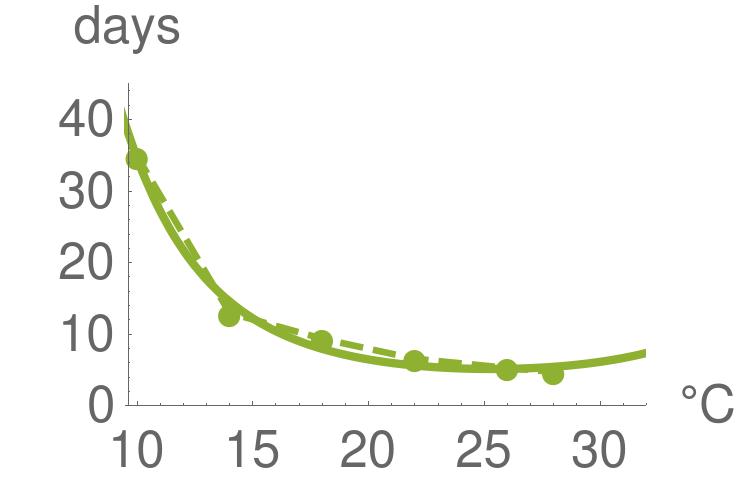 |
| --- | --- |
| Mean duration of egg stage | Mean duration of larva stage |
| 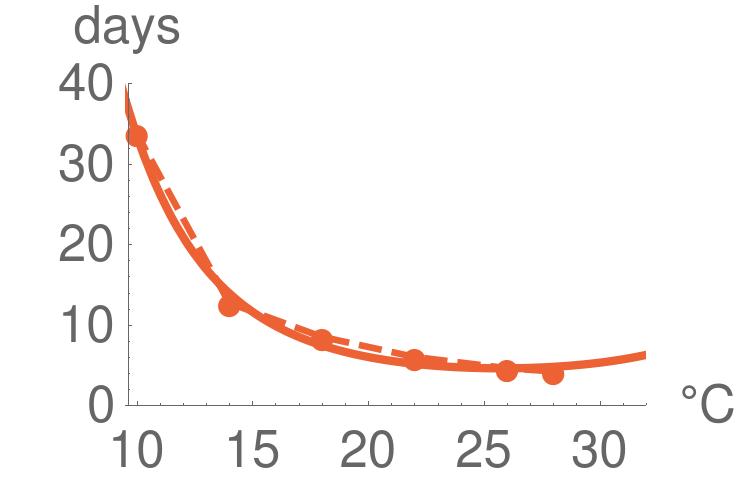 | 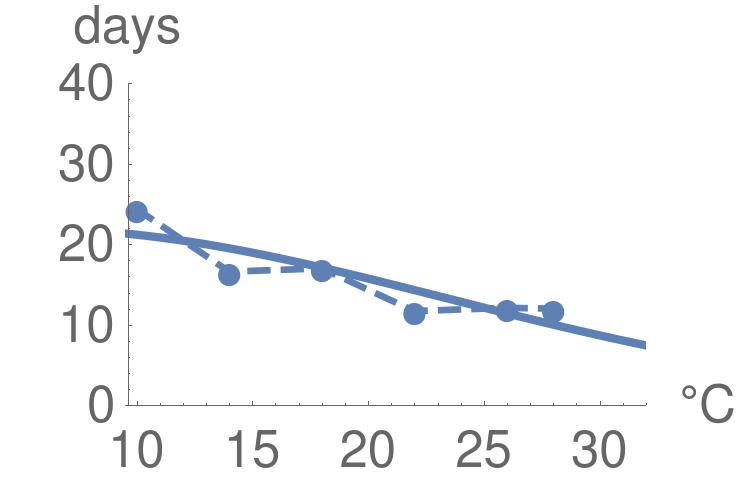 |
| Mean duration of pupal stage | Mean duration of adult stage |
| 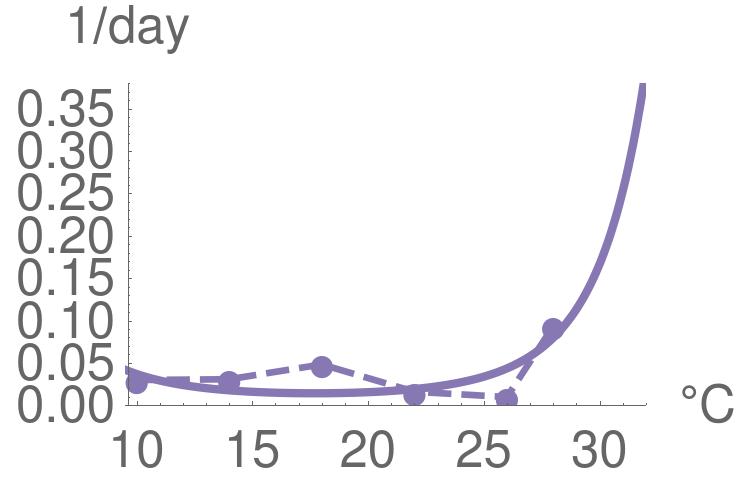 | 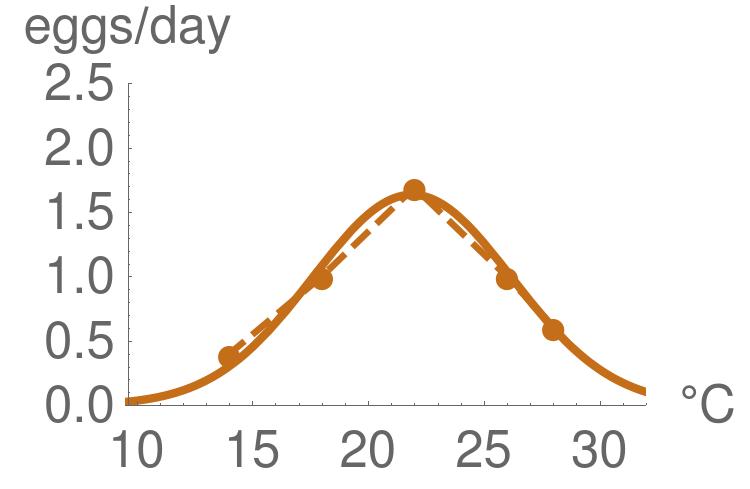 |
| Mean mortality during juvenile stages | Daily fecundity of female adults |

**Figure 1**. Temperature dependent properties.

All parameter values are summarized in Table 1.

Fecundity is (whereby we accounted for the sex ratio of ½)

$$\beta\left( w \right)=\frac{1}{2}f\left( c_{\beta},\mu_{\beta},\sigma_{\beta};w \right)$$

Other than in (5) we assume no competition for fruit, which could affect fecundity and juvenile mortality.

Average maturation delays for stage X=E, L and $P$ are

$$\Omega_{X}(w)=g\left( c_{X},\mu_{X},\sigma_{X};w \right)$$

Adult life length is

$$\Omega_{A}(w)=f\left( c_{A},\mu_{A},\sigma_{A};w \right)$$

where we assumed that the optimal temperature is $c_{A}=5$ °C to obtain a reasonable fit.

To estimate juvenile mortality, we used that the different treatments in (3) have been started with 100 individuals, and that the final sample sizes correspond to the number of surviving juveniles. We used the data on females and males because the resulting curves seemed more consistent. We combined the survival probabilities together with the (average) stage durations at the corresponding temperatures to obtain the daily mortality rates

$$\delta(w)=g\left( c_{\delta},\mu_{\delta},\sigma_{\delta};w \right)$$

| 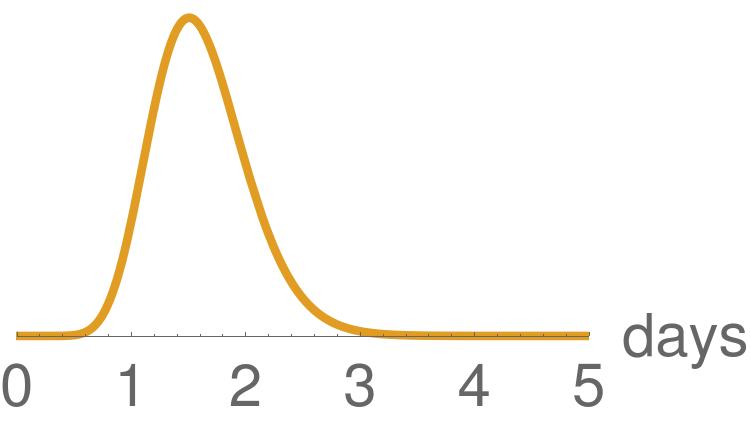 | 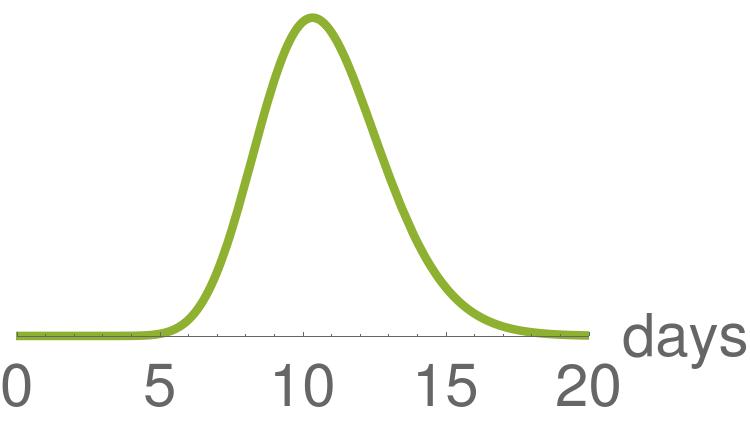 |
| --- | --- |
| Egg stage | Larval stage |
| 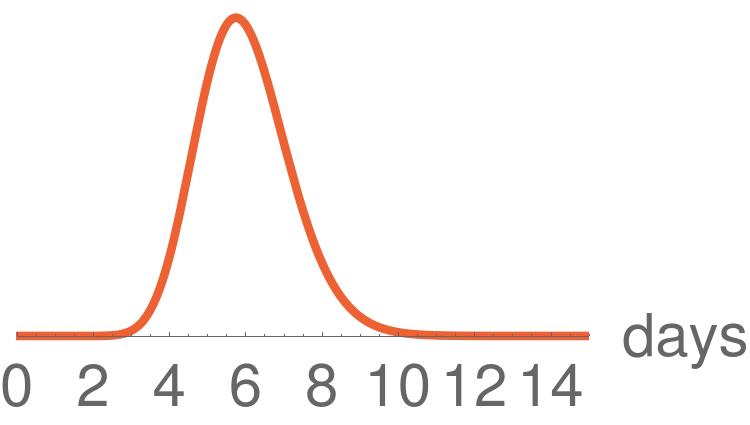 | 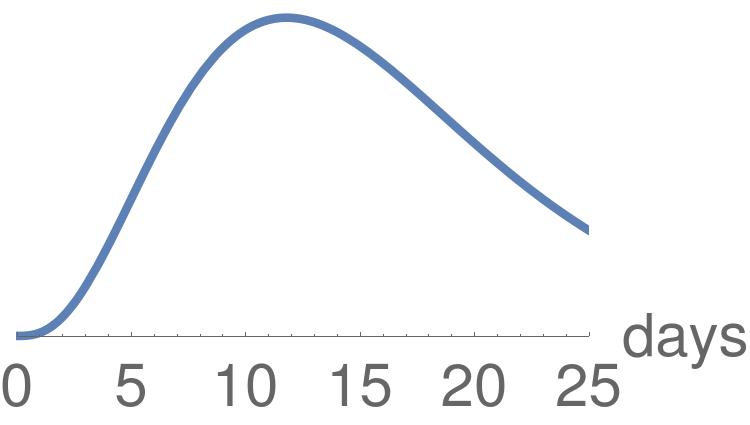 |
| Pupal stage | Adult stage |

Figure 2 Assumed distribution of stage durations around the mean duration at 20 °C.

For the modeling framework, we assumed that maturation delays are distributed randomly around the mean durations (at the given temperatures). We assumed that those distributions follow Gamma distributions, see Figure 2. This assumption allowed us to use the “linear chain trick” (6) and write out the system as a system of ordinary differential equations. For this, we subdivided each stage X=E, L, P and A into $k_{X}$ substages, see Figure 3.


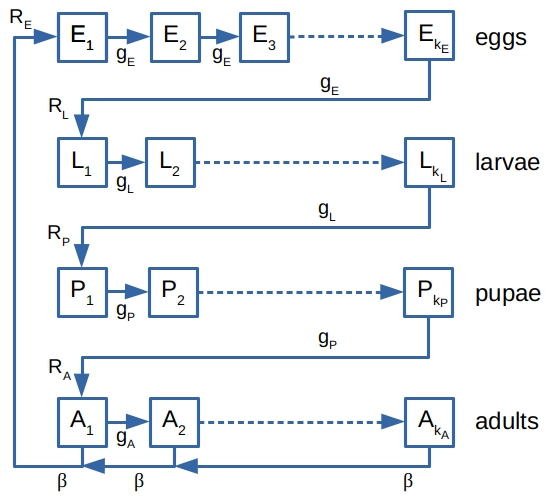


Figure 3 The principal stages E, L, P and A are subdivided into sub-stages to capture the stage durations

The speed with which a sub-stage of stage $X$ is left through maturation or aging (for juveniles and adults respectively) is

$$g_{X}\left( w \right)=k_{X}/\Omega_{X}\left( w \right)$$

The change in time of each sub-stage is

$$\frac{dX_{i}}{dt}=\left\{ \begin{matrix} R_{X} & i=1 \\ g_{X}\left( w \right) X_{i-1} & i>1 \end{matrix} \right.-X_{i} \left( g_{X}\left( w \right)+\left\{ \begin{matrix} \delta\left( w \right)X_{i} & X=E, L or P \\ 0 & X=A \end{matrix} \right. \right)$$

Here the first and the second term correspond to individuals entering and leaving the sub-stage respectively. The $R_{X}$ terms correspond to the rate with which the principal stages ($E$, $L$, $P$ and $A$) are entered. That is

$$R_{E}=\beta\left( w \right)A$$

$$R_{L}={g_{E}\left( w \right)E}_{k_{E}}$$

$$R_{P}={g_{L}\left( w \right)L}_{k_{L}}$$

$$R_{A}={g_{P}\left( w \right)P}_{k_{P}}$$

Table 1: Parameter values of the model.

| Parameter | Value | Description |
| --- | --- | --- |
| Fecundity | | |
| $c_{\beta}$ | 1.6 | Max daily fecundity, 1/day |
| $\mu_{\beta}$ | 21.9 | Optimal temp. for fecundity, °C |
| $\sigma_{\beta}$ | 6.1 | Temperature tolerance for fecundity, °C |
| Juvenile stage duration for eggs, larvae, and pupae ($X=E, L and P)$ | | |
| $c_{E},c_{L},c_{P}$ | 1.3, 5.1, 4.6 | Min duration, days |
| $\mu_{E},\mu_{L},\mu_{P}$ | 25.3, 25.3, 25.8 | Optimal temperature, °C |
| $\sigma_{E},\sigma_{L},\sigma_{P}$ | 11.0, 11.0, 11.2 | Temperature tolerance, °C |
| $k_{E},k_{L},k_{P}$ | 15, 25, 25 | Number of sub-stages (narrowness of variance) |
| Adult stage duration | | |
| $c_{A}$ | 22.0 | Max duration, days |
| $\mu_{A}$ | 5.0^[[1]](#footnote-2)^ | Optimal temperature, °C |
| $\sigma_{A}$ | 25.9 | Temperature tolerance, °C |
| $k_{A}$ | 4 | Number of sub-stages (narrowness of variance) |
| Juvenile mortality | | |
| $c_{\delta}$ | 0.014 | Baseline mortality, 1/day |
| $\mu_{\delta}$ | 17.7 | Optimal temperature, °C |
| $\sigma_{\delta}$ | 7.8 | Temperature tolerance, °C |

**Temperature**

We used data on mean daily temperature recorded at Aurora, Oregon, USA, between June and September 2020, see Figure 4.


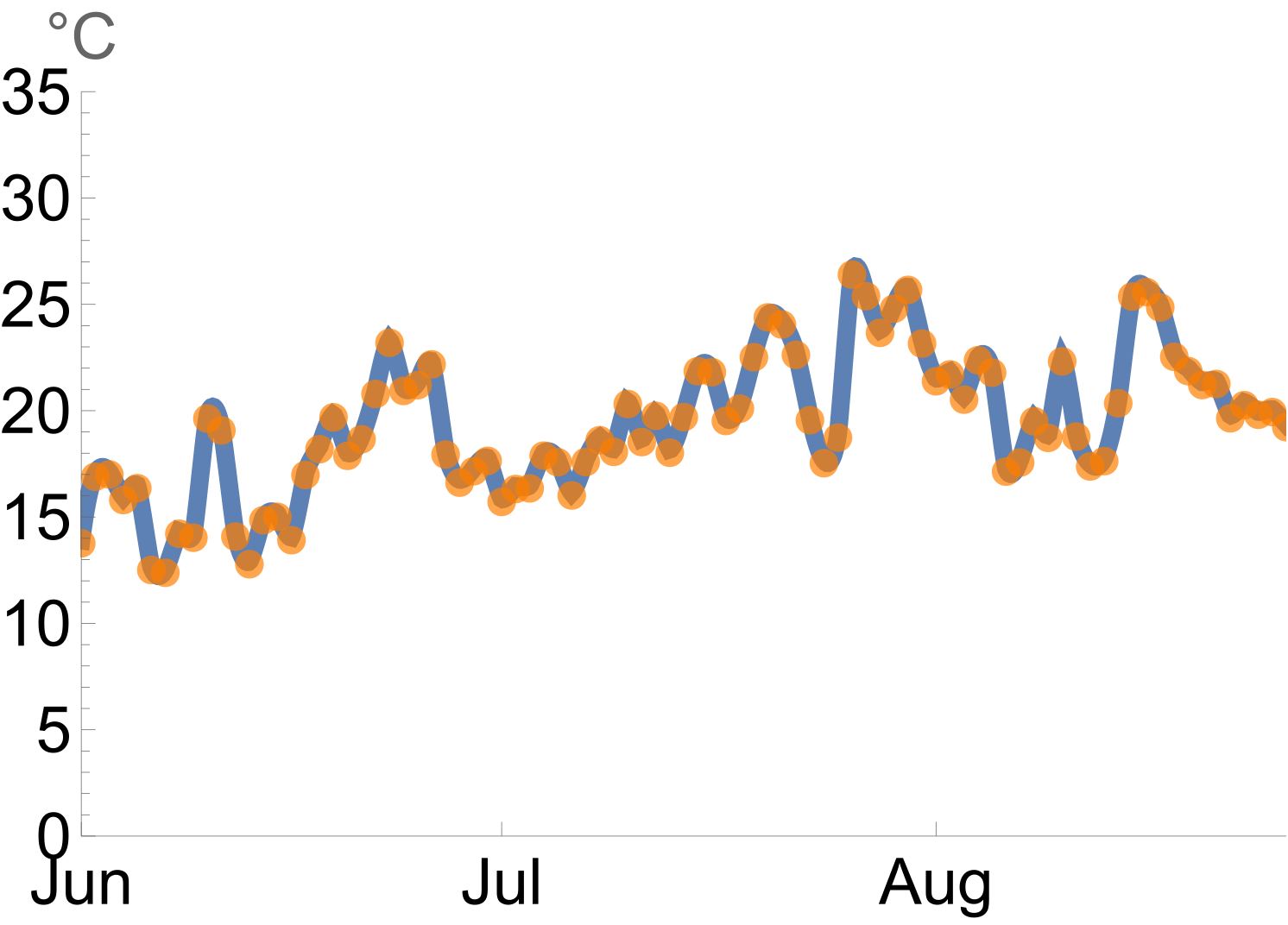


Figure 4 Temperature data used for the simulations.

**Insecticide mortalities**

We calculated insecticide induced mortalities rates caused by GS (spinosad) from laboratory data (2). The reported mortality probabilities when treated with a typical dose of spinosad over 6 h are for the different life stages as follows

| stage | mortality probability |
| --- | --- |
| egg | $82.8\%$ |
| larva | $75.9\%$ |
| pupa | $99.7\%$ |
| adult | $94.8\%$ |

The half-life decay time of the insecticide was reported to be $\lambda=$ 0.37 days (7). We assumed that the insecticide leads to the highest instantaneous mortality right after its application, and that its effect then diminishes exponentially. This means the decay rate $\delta$ is defined through $\frac{1}{2}=e^{\delta\lambda}$, i.e. $\delta=\frac{Log\left( 2 \right)}{\lambda}$. Using those quantities, the instantaneous mortality rates right after application, $\mu$, are defined through the equation $e^{-\mu\int_{0}^{6h} e^{-\delta t}dt}=1-p$. This equation can be solved, yielding $\mu=-\frac{\delta Log\left( 1-p \right)}{1-e^{-\delta6h}}$. For the simulations, we assumed that the insecticide was applied every 3 days, meaning that the mean dose in the field was $m=\frac{1}{3 day}\int_{0}^{\infty} e^{-\delta t}dt=0.18$ (relative to the dose right after application). To account for larger dissipation and uneven insecticide concentrations in the field, we reduced the insecticide-induced mortalities to $\sigma=$ 2% of their original values. This value was reported by Mermer et al. (under submission), who combined the same type of laboratory survival arrays with semi-field experiments for the population dynamics. In this way, the final insecticide induced mortality rates are given by $\tilde{\mu}=\sigma m \mu$ for each life stage, i.e.

| stage | Extra mortality rate in field |
| --- | --- |
| egg | $\tilde{\mu_{E}}=0.031/day$ |
| larva | $\tilde{\mu_{L}}=0.025/day$ |
| pupa | $\tilde{\mu_{P}}=0.10/day$ |
| adult | $\tilde{\mu_{A}}=0.05/day$ |

During inseciticide treatment, those mortality rates are added as follows

$$\frac{dX_{i}}{dt}=\ldots-{\tilde{\mu_{X}} X}_{i}$$

Where again, $X$ are the principal stages ($E$, $L$, $P$ and $A$), and $i$ is the subscript for the substages. The simulations were implemented with Wolfram Mathematica 13.0 (8). The code for the simulations is available online^[[2]](#footnote-3)^.

Gum application

Tait et al. (9) reported in semi-field conditions on blueberries that oviposition in the vicinity of GUM (solid gum) is reduced to $\gamma={14}/{28.7}=51\%$ of the value in control treatments without GUM (by releasing a certain number of *D. suzukii* in meshed enclosures with fruit plants and counting the number of eggs laid after a defined period of time). We used this factor to reduce fecundity in the model during GUM application, i.e. the rate of eggs recruited in the population is reduced to

$$R_{E}=\gamma\beta\left( w \right)A$$

**REFERENCE**

1. Mermer S, Pfab F, Hoheisel GA, Bahlol HY, Khot L, Dalton DT, et al. Canopy spray deposition and related mortality impacts of commonly used insecticides on *Drosophila suzukii* Matsumura (Diptera: Drosophilidae) populations in blueberry. Pest Management Science. 2020;76(4):1531–40.

2. Mermer S, Pfab F, Tait G, Isaacs R, Fanning PD, Van Timmeren S, et al. Timing and order of different insecticide classes drive control of *Drosophila suzukii*; a modeling approach. Journal of Pest Science. 2021;94(3):743–55.

3. Tochen S, Dalton DT, Wiman N, Hamm C, Shearer PW, Walton VM. Temperature-related development and population parameters for *Drosophila suzukii* (Diptera: Drosophilidae) on cherry and blueberry. Environmental Entomology. 2014;43(2):501–10.

4. Emiljanowicz LM, Ryan GD, Langille A, Newman J. Development, reproductive output and population growth of the fruit fly pest *Drosophila suzukii* (Diptera: Drosophilidae) on artificial diet. J Econ Entomol. 2014 Aug;107(4):1392–8.

5. Pfab F, Stacconi MVR, Anfora G, Grassi A, Walton V, Pugliese A. Optimized timing of parasitoid release: a mathematical model for biological control of *Drosophila suzukii*. Theor Ecol. 2018 Dec 1;11(4):489–501.

6. Diekmann O, Metz JAJ, Sabelis MW. Reflections and calculations on a prey-predator-patch problem. Acta Appl Math. 1989 Jan 1;14(1):23–35.

7. Abdelfatah RM, Saleh AA, Elgohary Laila RA, Negm SE. Dissipation of some Pesticide Residues in Tomato (*Lucopersicon esculentum L.*) Fruits Using QuECHERS Methodology under the Egyptian Field Conditions. Journal of Plant Protection and Pathology. 2020 Jul 1;11(7):327–32.

8. Latest Features in Mathematica 13 [Internet]. [cited 2022 Dec 27]. Available from: https://www.wolfram.com/mathematica/new-in-13/

9. Tait G, Kaiser C, Stacconi R, Dalton DT, Anfora G, Walton VM. A food-grade gum as a management tool for *Drosophila suzukii*. 2018; Bull of Insect 71 (2): 295-307.

1. We assumed that the optimal temperature for adult life length is $\mu_{A}=5$ °C to obtain a reasonable fit. [↑](#footnote-ref-2)
2. https://github.com/ferdi-p/gum-simulation-2022 [↑](#footnote-ref-3)
